# Fibroblast Growth Factor-23 and Risk of Cardiovascular Diseases: A Mendelian Randomization Study

**DOI:** 10.2215/CJN.05080422

**Published:** 2022-01-18

**Authors:** Killian Donovan, William G. Herrington, Guillaume Paré, Marie Pigeyre, Richard Haynes, Rebecca Sardell, Adam S. Butterworth, Lasse Folkersen, Stefan Gustafsson, Qin Wang, Colin Baigent, Anders Mälarstig, Michael V. Holmes, Natalie Staplin

**Affiliations:** 1 Clinical Trial Service Unit and Epidemiological Studies Unit, Nuffield Department of Population Health (NDPH), University of Oxford, Oxford, United Kingdom; 2 Medical Research Council Population Health Research Unit at the University of Oxford, NDPH, Oxford, United Kingdom; 3 Oxford Kidney Unit, Churchill Hospital, Oxford, United Kingdom; 4 Population Health Research Institute, McMaster University, Hamilton, Canada; 5 BHF Cardiovascular Epidemiology Unit, Department of Public Health and Primary Care, University of Cambridge, Cambridge, United Kingdom; 6 Danish National Genome Center, Copenhagen, Denmark; 7 Department of Medical Sciences, Molecular Epidemiology and Science for Life Laboratory, Uppsala University, Uppsala, Sweden; 8 Systems Epidemiology, Baker Heart and Diabetes Institute, Melbourne, Victoria, Australia; 9 Department of Medical Epidemiology and Biostatistics, Karolinska Institute, Solna, Sweden; 10 Big Data Institute, Li Ka Shing Centre for Health Information and Discovery, University of Oxford, Oxford, United Kingdom

## Abstract

**Background:**

Fibroblast growth factor-23 (FGF-23) is associated with a range of cardiovascular and noncardiovascular diseases in conventional epidemiological studies, but substantial residual confounding may exist. Mendelian randomization approaches can help control for such confounding.

**Methods:**

SCALLOP Consortium data of 19,195 participants were used to generate an FGF-23 genetic score. Data from 337,448 UK Biobank participants were used to estimate associations between higher genetically predicted FGF-23 concentration and the odds of any atherosclerotic cardiovascular disease (*n*=26,266 events), nonatherosclerotic cardiovascular disease (*n*=12,652), and noncardiovascular diseases previously linked to FGF-23. Measurements of carotid intima-media thickness and left ventricular mass were available in a subset. Associations with cardiovascular outcomes were also tested in three large case-control consortia: CARDIOGRAMplusC4D (coronary artery disease, *n*=181,249 cases), MEGASTROKE (stroke, *n*=34,217), and HERMES (heart failure, *n*=47,309).

**Results:**

We identified 34 independent variants for circulating FGF-23, which formed a validated genetic score. There were no associations between genetically predicted FGF-23 and any of the cardiovascular or noncardiovascular outcomes. In UK Biobank, the odds ratio (OR) for any atherosclerotic cardiovascular disease per 1-SD higher genetically predicted logFGF-23 was 1.03 (95% confidence interval [95% CI], 0.98 to 1.08), and for any nonatherosclerotic cardiovascular disease, it was 1.01 (95% CI, 0.94 to 1.09). The ORs in the case-control consortia were 1.00 (95% CI, 0.97 to 1.03) for coronary artery disease, 1.01 (95% CI, 0.95 to 1.07) for stroke, and 1.00 (95% CI, 0.95 to 1.05) for heart failure. In those with imaging, logFGF-23 was not associated with carotid or cardiac abnormalities.

**Conclusions:**

Genetically predicted FGF-23 levels are not associated with atherosclerotic and nonatherosclerotic cardiovascular diseases, suggesting no important causal link.

**Podcast:**

This article contains a podcast at https://dts.podtrac.com/redirect.mp3/www.asn-online.org/media/podcast/CJASN/2023_01_10_CJN05080422.mp3

## Visual Abstract

**Figure:**
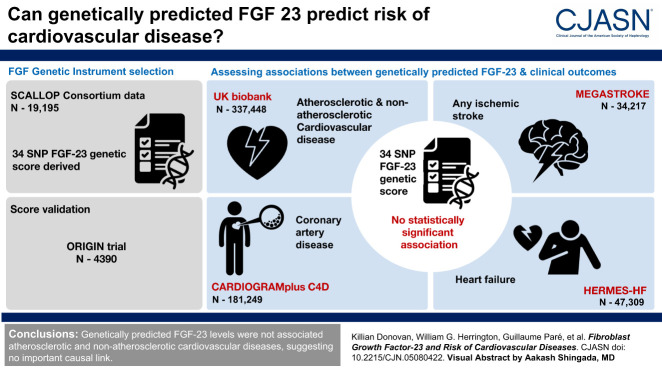

## Introduction

Higher risk of cardiovascular disease emerges early in the development of chronic kidney disease (CKD), and the risk is progressively higher as kidney function declines.^1,2^ Arterial disease in advanced CKD exhibits nonatheromatous noncalcified arterial stiffening, intimal atherosclerotic lesions, and heavy medial calcification.^3^ Correspondingly, CKD is associated with both structural and coronary heart disease. Perhaps about one-half of this risk is explained by the effects of CKD on blood pressure.^3^ Nontraditional risk factors associated with dysregulated phosphate and calcium homeostasis may also be important.^4–6^

Fibroblast growth factor-23 (FGF-23) is a hormonal promoter of urinary phosphate excretion, increasing in blood concentration in early CKD.^7^ The actions of FGF-23 are generally limited to tissues where the coreceptor Klotho is expressed, and particularly in the renal tubules where it downregulates sodium-phosphate cotransporters.^8,9^ Animal studies suggest direct (*i.e.*, Klotho-independent effect) cardiotoxicity,^10^ leading to hypotheses that FGF-23 should be considered not just as a marker for cardiovascular disease but also as a causal contributory factor.^11^ Meta-analysis of conventional epidemiological studies has found independent associations between higher circulating FGF-23 concentration with higher risk of atherosclerotic cardiovascular diseases (*i.e.*, myocardial infarction and stroke) and heart failure.^12^ However, substantial uncertainty about causality remains as these associations do not exhibit a clear “exposure-response” relationship and are nonspecific: Positive associations between FGF-23 and risks of infection,^13^ fractures,^14^ acute kidney injury (AKI),^15^ and all-cause mortality^16^ are also reported. Residual confounding therefore remains a possible explanation for these FGF-23 associations.

Naturally occurring genetic variants (single nucleotide polymorphisms [SNPs]) associated with biological traits are allocated randomly at conception and can be used as instruments in genetic epidemiological analyses. This Mendelian randomization (MR) approach can avoid some of the limitations inherent to conventional observational studies^17–19^ and has a particular advantage when aiming to control for confounding by kidney function. Previously reported MR studies of FGF-23 have been limited by low power as the genetic variants used explain only approximately 3% of the variation in FGF-23, and they have not explored the breakdown of associations with atherosclerotic versus nonatherosclerotic phenotypes.^20–22^ We aimed to derive a more powerful genetic score for FGF-23 from a large international collaboration's genotypic and proteomic data and then use it to estimate associations between lifelong genetically predicted differences in circulating FGF-23 with risk of cardiovascular diseases in the UK Biobank cohort, and in the CARDIOGRAMplusC4D, MEGASTROKE, and HERMES-HF case-control consortia. We considered genetic associations for atherosclerotic and nonatherosclerotic cardiovascular phenotypes separately, and a range of noncardiovascular diseases identified in nongenetic epidemiological studies.

## Methods

### Study Populations and Data

Table 1 summarizes the study design and the different study populations used to derive and validate the novel FGF-23 genetic score and then test associations with clinical outcomes and measurements. The score was derived and validated in cohorts with a low CKD prevalence to reduce the risk of identifying pleiotropic variants associated with FGF-23 only through their association with kidney disease.

**Table 1:** Study design and sources of data

Step	Genotyped Data Source	Design	Outcome	Sample Size
1. Identify SNPs associated with FGF-23 and derive SNP-specific weights	SCALLOP^23^	GWAS	FGF-23 measurements	19,195
2. Validate genetic score	ORIGIN^24^	Ordinal regression model	FGF-23 measurements	4390
3. Assess genetic associations between FGF-23 with clinical outcomes or measurements	UK Biobank^25^ (*n*=337,448)		Clinical outcomes:	Participants with outcome
		34-SNP genetic score and logistic regression	Atherosclerotic cardiovascular diseases	26,266
			Nonfatal myocardial infarction	9677
			Coronary revascularization	14,646
			Coronary death	2258
			Ischemic stroke	5992
			Other revascularization	3782
		34-SNP genetic score and logistic regression	Nonatherosclerotic cardiovascular diseases	12,652
			Hospitalization with heart failure	10,177
			Noncoronary cardiac death	689
			Other vascular death	617
			Hemorrhagic stroke	1745
		34-SNP genetic score and logistic regression	Selected noncardiovascular diseases	
			Any fracture	51,166
			Fragility fracture	6624
			Hospitalization for infection	38,613
			Hospitalization with AKI	11,569
			End-stage kidney disease	774
			Noncardiovascular death	17,196
		34-SNP genetic score and linear regression	Clinical imaging measurements:	
			Carotid intima-media thickness	31,461
			Left ventricular mass index	18,710
			Bone mass	3695
			Bone mineral density	3704
				Cases/controls
	CARDIOGRAMplusC4D^26^	Inverse-variance weighted two-sample MR	Coronary artery disease	181,249/984,401
	MEGASTROKE^27^		Any ischemic stroke	34,217/406,111
	HERMES-HF^28^		Heart failure	47,309/930,014

SNP, single nucleotide polymorphism; FGF-23, fibroblast growth factor-23; GWAS, genome-wide association study.

SCALLOP Consortium data from 19,195 individuals were used to identify variants associated with FGF-23 for a novel genetic score. SCALLOP is a collaboration of genotyped cohorts with proteomic measurements using the multiplex immunoassay Olink platform.^23^ The assay uses two antibodies, which separately bind at FGF-23's N-terminus and C-terminus, analogous to intact FGF-23 assays.

Once derived, the genetic score was validated in an independent cohort of 4390 genotyped individuals from the ORIGIN trial in people with dysglycaemia whose blood had also been assayed for FGF-23 using a Luminex platform^24^ (Myriad-RBM).

Associations between the FGF-23 genetic score and risk of clinical outcomes and measurements were first assessed in UK Biobank, a prospective genotyped cohort of 502,650 UK adults aged 40–69 years recruited between 2006 and 2010.^25^ UK Biobank data include self-completed touch-screen questionnaires, computer-assisted interviews, physical and functional measurements, and biochemical assays. Genotyping was performed using the Affymetrix UK BiLEVE Axiom array and the Affymetrix UK Biobank Axiom array, with imputation with IMPUTE4 using the Haplotype Reference Consortium and the UK 10K and 1000 Genomes phase 3 reference panels.^29^ UK Biobank has been linked to routinely collected UK mortality and hospital admission data from 1998 from which a range of clinical outcomes can be derived. The present analyses included unrelated White British participants with available genetic data meeting quality control standards (*n*=335,536) and excluded those who had withdrawn their data from the UK Biobank (*n*=88) or had missing genetic data. A subset of these participants has also undergone carotid ultrasound imaging (*n*=31,461 were included in this study), cardiac magnetic resonance imaging (*n*=18,734), and DEXA scanning of bone mass (*n*=3695).

Associations between FGF-23 with risk of specific cardiovascular outcomes were also assessed in three large case-control consortia: CARDIOGRAMplusC4D,^26^ MEGASTROKE,^27^ and HERMES.^28^ These international case-control consortia comprise genomic and clinical outcome data for individuals with coronary artery disease (181,249 cases/984,401 controls), ischemic stroke (34,217 cases/406,111 controls), and heart failure (47,309 cases/930,014 controls), respectively.

### FGF-23 Genetic Instrument Selection

A genome-wide association study (GWAS) was performed within European ancestry cohorts participating in SCALLOP using additive models adjusted for age, sex, and population structure. Autosomal variants associated with FGF-23 at *P*<5×10^−6^ in a meta-analysis across the SCALLOP Consortium were clumped using PLINK^30^ to a list of 34 lead variants separated by at least 1000 kb and with *R*^2^<0.1. A genetic score for individual ORIGIN and UK Biobank participants was constructed with a weighted sum of the dosages of these SNPs using the SCALLOP estimates as weights for each variant. Associations between this score and measured FGF-23 levels were confirmed in the ORIGIN cohort.^24^ Any *cis*-variants within 100 kb of the FGF-23 transcription start site were also selected as instruments for sensitivity analyses.

### Outcomes

Study outcomes were selected based on those previously observed conventional observational associations with FGF-23.^12^ The key cardiovascular outcomes in UK Biobank were “any atherosclerotic cardiovascular disease,” a composite of coronary death, nonfatal myocardial infarction, ischemic stroke, or revascularization, and separately “any nonatherosclerotic cardiovascular disease,” a composite of noncoronary cardiac and other vascular death, hospitalization with heart failure, or hemorrhagic stroke. Noncardiovascular outcomes included any bone fracture, and the subset with a fragility fracture, hospitalization for infection, hospitalization with AKI, treated end-stage kidney disease, and any noncardiovascular death (see Supplemental Methods for code definitions). In the UK Biobank subset of participants with magnetic resonance imaging, left ventricular mass index was established using previously published algorithms.^31^ Mean and maximum carotid intima-media thickness were used among the subset with carotid ultrasound assessments. Android/gynoid bone mass and bone mineral density of lumbar vertebrae and the femoral neck were used as measurements of bone health.

### Statistical Analyses

The genetic score was validated by regression of measured FGF-23 on the genetic score in the ORIGIN cohort, adjusted for age, sex, and ethnicity. Ordinal regression was used because of a high proportion (59%) of participants in ORIGIN with FGF-23 levels below the lower limit of detection of the assay. Estimates of associations between binary clinical outcomes and the genetic score were ascertained from logistic regression models adjusted for age, sex, and the first 40 genomic principal components. Linear regression models, including the same covariates, were used for continuous outcomes. Analyses of case-control consortia data used a two-sample inverse variance weighted MR method with summary data for the 34 SNPs (SNP-logFGF23 associations from SCALLOP and SNP outcome associations from relevant consortia). For the key analyses of cardiovascular and noncardiovascular outcomes, a significance level of *P*<0.05 was used for all individual statistical tests. For the subsidiary assessments using imaging-based clinical measurements and for sensitivity analyses, a Bonferroni correction was applied to *P* values. UK Biobank participants who had withdrawn or were lost to follow-up were excluded from analyses, and only those with available genotyping data were included.

The key sensitivity analysis was to assess the association for the variants within 100 kb of the FGF-23 transcription start site (*i.e.*, any *cis-*variants), as such variants are less likely to have unidentified pleiotropic effects.^32^ We also performed sensitivity analyses excluding any potentially pleiotropic variants associated with estimated glomerular filtration rate (eGFR), body mass index, or cardiovascular risk factors other than FGF-23. Such variants were identified from published associations or UK Biobank using the PhenoScanner database.^33,34^ Any effects of residual linkage disequilibrium between selected variants or between “clumps” were assessed in a sensitivity analysis using a genetic score including only the single lead variant at each identified locus. Standard approaches to testing for violations of the instrumental variable assumptions were also conducted.^35–38^ Analyses were performed in SAS version 9.4 (SAS Institute, Cary, NC) and R v3.6.2.

## Results

### Genetic Instrument Derivation, Validation, and Power

Thirty-four independent variants associated at *P*<5×10^−6^ with circulating FGF-23 concentration were identified from SCALLOP Consortium data (see Supplemental Table 1 for details of each SNP). Genomic inflation in this GWAS was acceptable (*λ*=1.008; Supplemental Figure 1). Of the 34 variants, four were found to be associated with cardiovascular risk factors other than FGF-23 (see Supplemental Table 2), and two independent *cis-*variants were identified (rs6489536 [FGF-23 increasing allele: C], rs7955866 [FGF-23 increasing allele: G]; *R*^2^=0.08; Supplemental Table 3). The 34 SNPs collectively accounted for 6.3% of the variance in log-transformed FGF-23 (Supplemental Table 4), and the two *cis*-variants accounted for 0.4%.

### Validation of Genetic Variants

We replicated associations with four of five loci identified in a recent GWAS (5q35.3, 9q21.11, 9q34.2, and 20q13.2; Supplemental Tables 1 and 5).^21^ To further validate our identified associations, we sought comparable GWAS with available summary data. Two were identified (one of 900 Scottish adults using the Olink proteomic platform^39^ and one of 5000 healthy Icelanders using the SomaLogic platform).^40^ These studies were underpowered to identify FGF-23–associated variants, so we validated only *cis-*variants in these studies (Supplemental Table 5).

The SCALLOP, ORIGIN, and UK Biobank cohorts had similar distributions of effect alleles and similar ancestry (Supplemental Table 6). A higher genetic score was associated with higher measured FGF-23 in the independent ORIGIN study, validating the novel score (Supplemental Table 7). The genetic score and number of atherosclerotic and nonatherosclerotic cardiovascular outcomes in the included UK Biobank population provided 80% power at α=0.05 to detect a 1.08 and 1.11 minimum odds ratio (OR) per 1-SD higher logFGF-23, respectively (Supplemental Table 4).

### UK Biobank Population Characteristics

The median age among the 337,448 included UK Biobank participants was 58 (Q1–Q3: 51–63) years, 181,216 (54%) were female, 33,971 (10%) were current smokers, and 16,218 (5%) reported a history of diabetes mellitus. The mean (SD) body mass index was 27.4 (4.7) kg/m^2^, and the median eGFR was 91 (81–100) ml/min per 1.73 m^2^. There were no important differences in age, sex, lifestyle factors, anthropometric measurements, blood pressure, prevalence of diabetes mellitus, glycated hemoglobin levels, eGFR, and markers of calcium/phosphate homeostasis across fifths of the FGF-23 genetic score (Table 2).

**Table 2:** Baseline characteristics of included UK Biobank participant by fifths of the FGF-23 genetic score

Characteristic	All Participants (*n*=337,448)	Bottom Fifth of Score (*n*=67,489)	Middle Fifth of Score (*n*=67,489)	Top Fifth of Score (*n*=67,486)	Difference Between Top and Bottom Fifth
Age, yr	58 (51–63)	58 (51–63)	58 (51–63)	58 (51–63)	0
Female	181,216 (54)	36,288 (54)	36,266 (54)	36,310 (54)	0%
Current smoker	33,971 (10)	6694 (10)	6870 (10)	6753 (10)	0%
Body mass index (kg/m^2^)	27.4 (5)	27.4 (5)	27.4 (5)	27.4 (5)	0.0
Waist-to-hip ratio	0.87 (0.09)	0.87 (0.09)	0.87 (0.09)	0.87 (0.09)	0
Diabetes mellitus (self-report)	16,218 (5)	3332 (5)	3272 (5)	3214 (5)	0%
HbA1c (%)	5.4 (5.1–5.6)	5.4 (5.1–5.6)	5.4 (5.1–5.6)	5.4 (5.2–5.6)	0.0
**BP**					
Systolic (mm Hg)	138 (19)	138 (19)	138 (19)	138 (19)	0
Diastolic (mm Hg)	82 (10)	82 (10)	82 (10)	82 (10)	0
Antihypertensive use	78,433 (23%)	15,678 (23%)	15,790 (23%)	15,554 (23%)	0%
**Kidney function**					
eGFR (ml/min per 1.73 m^2^)	91 (81–100)	91 (81–100)	91 (81–100)	90 (81–100)	1
eGFR <45 or on KRT	1994 (<1)	392 (<1)	403 (<1)	421 (<1)	0%
eGFR ≥45	319,362 (95)	63,950 (95)	63,869 (95)	63,709 (94)	1%
eGFR missing	16,092 (5)	3147 (5)	3217 (5)	3356 (5)	0%
Urinary UACR >30 mg/g	14,303 (4)	2839 (4)	2857 (4)	2864 (4)	0%
**Cholesterol**					
LDL cholesterol (mg/dl)	135 (116–159)	135 (116–159)	135 (116–159)	135 (116–159)	0
HDL cholesterol (mg/dl)	54 (46–66)	54 (46–66)	54 (46–66)	54 (46–66)	0
**Markers of calcium/phosphate homeostasis**					
Calcium (mg/dl)	9.5 (0.4)	9.5 (0.4)	9.5 (0.4)	9.5 (0.4)	0.0
Phosphate (mg/dl)	3.6 (0.5)	3.6 (0.5)	3.6 (0.5)	3.6 (0.5)	0.0
Vitamin D (ng/ml)	49.8 (21.0)	49.4 (20.9)	49.9 (20.9)	50.1 (21.1)	0.7

Data are mean (SD), *n* (%), or median (Q1–Q3).

FGF-23, fibroblast growth factor-23; HbA1c, glycosylated hemoglobin; UACR, urinary albumin:creatinine ratio.

BP is the mean of two measurements. eGFR calculated by CKD-EPI combined creatinine+cystatin C equation (Inker *et al.* *N Engl J Med* 2012; 367:20-29, 2012).

### FGF-23 and Risk of Atherosclerotic Cardiovascular Disease

**Figure 1:**
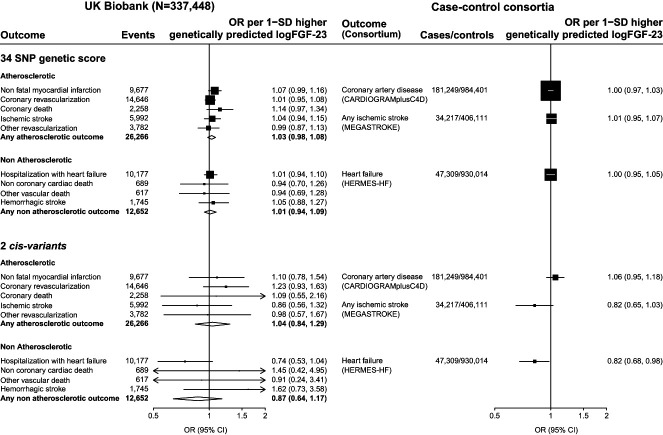
**Associations between genetically predicted FGF-23 with risk of cardiovascular outcomes**. FGF‐23, fibroblast growth factor 23; SNP, single nucleotide polymorphism; OR, odds ratio; 95% CI, 95% confidence interval.

In total, 26,266 (8%) UK Biobank participants had atherosclerotic cardiovascular disease. There was no association between genetically predicted FGF-23 with the composite of any atherosclerotic cardiovascular disease (OR per 1-SD higher logFGF-23=1.03; 95% confidence interval [95% CI], 0.98 to 1.08), nor were there any significant associations with any of its constituent components: nonfatal myocardial infarction (1.07; 95% CI, 0.99 to 1.16; 9677 outcomes), coronary death (1.14; 95% CI, 0.97 to 1.34; 2258 outcomes), ischemic stroke (1.04; 95% CI, 0.94 to 1.15; 5992 outcomes), coronary revascularization (1.01; 95% CI, 0.95 to 1.08; 14,646 outcomes), or other revascularization (0.99; 95% CI, 0.87 to 1.13; 3782 outcomes; Figure 1).

Analyses of 1.17 million participants in CARDIoGRAMplusC4D found no significant association between genetically predicted FGF-23 and coronary artery disease (OR per 1-SD higher genetically predicted log[FGF-23] 1.00; 95% CI, 0.97 to 1.03; 181,249 cases). In the European ancestry subset of the MEGASTROKE consortium (440,328 participants), 30 of the FGF-23 SNPs were available (missing SNPs: rs11542063, rs117612483, rs117989952, and rs189972262 with no available strong proxies). Inverse variance weighted MR also demonstrated no significant association between genetically predicted FGF-23 with risk of ischemic stroke (OR per 1-SD higher genetically predicted log[FGF-23] 1.01; 95% CI, 0.95 to 1.07; 34,217 cases: Figure 1).

Among the subset of 31,461 UK Biobank participants with carotid imaging, there was no significant association between genetically predicted FGF-23 and carotid intima-media thickness. A 1-SD higher genetically predicted FGF-23 was associated with a −1-μm difference in mean carotid intima-media thickness (95% CI, −6 to 4 μm) and a 0-μm difference in maximum carotid intima-media thickness (95% CI, −6 to 6 μm: Table 3).

**Table 3:** Association of genetically predicted FGF-23 with clinical measurements

Clinical Measurement	Number with Measurement	Median (Q1–Q3)	Estimated Effect of 1-SD Higher logFGF-23 (95% CI)	Bonferroni Corrected *P* Value^a^
**Carotid intima-media thickness, in μm**				
Mean	31,461	669 (596–760)	−1 (−6 to 4)	0.99
Maximum	31,461	780 (693–885)	0 (−6 to 6)	0.99
Left ventricular mass index, in g/m^2^	18,710	44.0 (38.9–50.4)	0.4 (−0.0 to 0.7)	0.33
**Bone mass, in g**				
Android	3695	48 (40–57)	1 (0 to 3)	0.45
Gynoid	3695	267 (221–324)	9 (3 to 15)	0.01
**Bone mineral density, in mg/cm^3^**				
Lumbar vertebrae	3679	1184 (1056–1319)	24 (0 to 48)	0.30
Femoral neck	3704	945 (852–1046)	17 (0 to 35)	0.29

Mean carotid intimal-media thickness is calculated by the mean of four measurements, two on the left and two on the right. Maximum carotid intima-medial thickness is the maximum of these four values.

FGF-23, fibroblast growth factor-23; 95% CI, 95% confidence interval.

a *P* values are Bonferroni adjusted for the seven clinical measurement outcomes. In sensitivity analyses, bone mass and bone mineral density (BMD) associations were attenuated on exclusion of variants with possible effects on vitamin D metabolism—effect per 1-SD higher genetically predicted FGF-23 (95% CI, Bonferroni-corrected *P*) for android bone mass 1 g (95% CI, 0 to 3, *P*=0.63), gynoid bone mass 7 g (95% CI, 2 to 14, *P*=0.09), lumbar BMD 22 mg/cm^3^ (95% CI, −4 to 49, *P*=0.51), and femoral neck BMD 12 mg/cm^3^ (95% CI, −7 to 30, *P*=0.84).

### FGF-23 and Risk of Nonatherosclerotic Cardiovascular Disease

In total, 12,652 (4%) UK Biobank participants had nonatherosclerotic cardiovascular disease. There was no association between genetically predicted FGF-23 with any nonatherosclerotic cardiovascular disease (OR per 1-SD higher logFGF23=1.01; 95% CI, 0.94 to 1.09). This included no significant association with its constituent components: hospitalization with heart failure (1.01; 95% CI, 0.94 to 1.10; 10,177 outcomes), noncoronary cardiac death (0.94; 95% CI, 0.70 to 1.26; 689 outcomes), noncardiac vascular death (0.94; 95% CI, 0.69 to 1.28; 617 outcomes), or hemorrhagic stroke (1.05; 95% CI, 0.88 to 1.27; 1745 outcomes; Figure 1).

Among 977,323 participants in HERMES-HF, 29 of the FGF-23–associated variants identified in SCALLOP were available (missing SNPs: rs11542063, rs117612483, rs117989952, rs189972262, and rs75357988, with no available strong proxies). Inverse variance weighted MR using data for the remaining 29 SNPs found no significant association between genetically predicted FGF-23 with heart failure (OR per 1-SD higher genetically predicted log[FGF-23] 1.00; 95% CI, 0.95 to 1.05; 47,309 cases; Figure 1).

Among the UK Biobank subset with cardiac magnetic resonance imaging, the median (Q1–Q3) left ventricular mass index (*i.e.*, left ventricular mass per square meter of body surface area) was 44.0 g/m^2^ (95% CI, 38.9 to 50.4). There was no significant association between genetically predicted FGF-23 and left ventricular mass index (estimated difference in left ventricular mass index per 1-SD higher genetically predicted logFGF-23 was 0.4 g/m^2^ [95% CI, –0.0 to 0.7; Table 3]).

### FGF-23 and Risk of Noncardiovascular Outcomes

**Figure 2:**
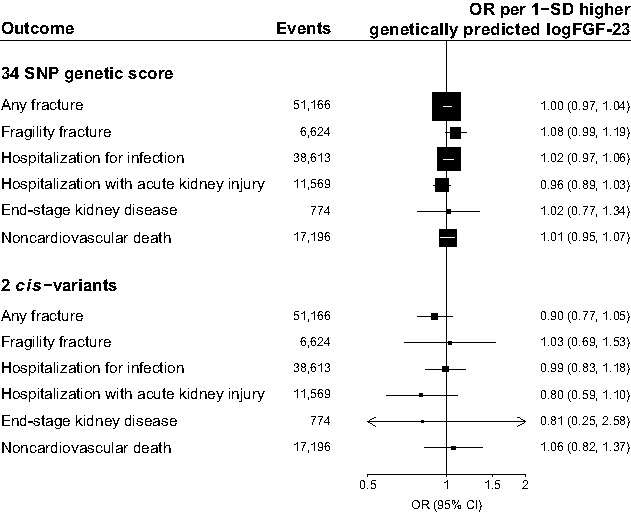
**Associations between genetically predicted FGF-23 with risk of noncardiovascular outcomes**. FGF‐23, fibroblast growth factor 23; SNP, single nucleotide polymorphism; OR, odds ratio; 95% CI, 95% confidence interval.

In UK Biobank data, there was no significant association between genetically predicted FGF-23 with risk of any of the noncardiovascular outcomes. The ORs per 1-SD higher genetically predicted logFGF-23 were 1.00 for any fracture (95% CI, 0.97 to 1.04; *n*=51,166), 1.08 for any fragility fracture (95% CI, 0.99 to 1.19; *n*=6624), 1.02 for hospitalization for infection (95% CI, 0.97 to 1.06; *n*=38,613), 0.96 for hospitalization with AKI (95% CI, 0.89 to 1.03; *n*=11,569), 1.02 for treated end-stage kidney disease (95% CI, 0.77 to 1.34; *n*=774), and 1.01 for any noncardiovascular death (95% CI, 0.95 to 1.07; *n*=17,196 outcomes; Figure 2).

Higher genetically predicted FGF-23 concentrations were associated with higher gynoid (pelvic girdle) bone mass but not android (lumbar) bone mass, nor bone mineral density in the lumbar vertebrae or femoral neck (Table 3). These associations were no longer significant in a sensitivity analysis excluding four SNPs at the *CYP24A1* locus (rs13038432, rs2870308, rs290403, and rs1570669), as their associations with FGF-23 may be indirectly mediated *via* effects on vitamin D metabolism (Table 3). These associations were also not apparent when using the *cis*-variants alone.

### Sensitivity Analyses

Analyses using two *cis-*variants had limited power in UK Biobank (minimum detectable OR with 80% power and *α*=0.05 for any atherosclerotic event=1.47 per 1-SD higher genetically predicted logFGF-23). Inverse variance weighted MR using these variants yielded an estimated OR per 1-SD higher logFGF-23 of 1.04 (95% CI, 0.84 to 1.29) for any atherosclerotic and 0.87 (95% CI, 0.64 to 1.17) for any nonatherosclerotic cardiovascular outcome (Figure 1). Two-sample MR analyses using the case-control consortia provided more power. The minimum detectable ORs for coronary artery disease, stroke, and heart failure per FGF-23 increasing allele were 1.13, 1.26, and 1.18, respectively. In such analyses, there was no association of genetically predicted FGF-23 with higher risk of coronary artery disease (1.06; 95% CI, 0.95 to 1.18), ischemic stroke (0.82; 95% CI, 0.65 to 1.03), or heart failure (0.82; 95% CI, 0.68 to 0.98; Figure 1). The findings were robust to a series of other sensitivity analyses using different MR methods and excluding potentially pleiotropic variants (Supplemental Figures 2–5; Supplemental Tables 8–13). No significant heterogeneity in SNP effects was observed for key clinical outcomes (Supplemental Figure 6).

## Discussion

Our overriding aim was to conduct powerful and unconfounded genetic analyses capable of detecting the size of associations between FGF-23 and cardiovascular diseases, which have been reported in conventional epidemiological studies.^12^ This was achieved by deriving a novel FGF-23 genetic score from approximately 19,000 individuals who have contributed to a large collaborative consortium of studies with genomic and proteomic data and then performing genetic analyses in large genotyped datasets, including UK Biobank and case-control consortia, which provided data on approximately 180,000 coronary disease cases, approximately 35,000 strokes, and approximately 50,000 people with heart failure. Our novel 34-variant FGF-23 genetic score was validated and found to be unconfounded with respect to key confounders, including kidney function. A range of subsequent genetic analyses found no significant associations between genetically predicted FGF-23 with risk of coronary artery disease, ischemic stroke, or heart failure, nor were there any significant associations with clinical measurements of carotid artery atherosclerosis or imaging evidence of structural heart disease, indicating that the FGF-23 molecule is unlikely to have a direct causal role in the pathogenesis of cardiovascular diseases.

Observational studies in general populations have found a higher risk of myocardial infarction, stroke, and heart failure in the order of 40% per 20 pg/ml higher FGF-23 concentration (about 1-SD in general populations).^12,41,42^ Used within the available outcome datasets in this report, our FGF-23 genetic instrument was estimated to have 80% power to detect true higher odds of cardiovascular diseases in the order of 5%–15%. The lack of any significant genetic FGF-23-cardiovascular disease associations in the presented analyses suggests that, if a causal relationship between FGF-23 and any cardiovascular disease does exist, its size is likely to be substantially smaller than that observed in conventional epidemiological studies. We also did not find any human evidence to support the findings from animal studies that FGF-23 stimulates left ventricular hypertrophy.^43^ Our analyses also had reasonable power to test hypotheses about FGF-23 and the risk of certain noncardiovascular diseases. Contrary to findings from conventional epidemiological studies, we found no evidence for associations between genetically predicted FGF-23 and risk of infection,^13^ fractures,^14^ or AKI.^15^

A prior MR study of FGF-23 and cardiovascular outcomes using publicly available summary statistics reported a protective effect of FGF-23 against coronary artery disease (*i.e.*, the opposite association to conventional analyses).^44^ This study and another recent study have found no effect on heart failure.^45^ The genetic instrument in the previous studies had more limited power. This study benefits from the large scale of data from the SCALLOP Consortium,^23^ UK Biobank,^25^ and the case-control consortia providing a novel, more powerful genetic instrument, including *cis*-variants and a wider range of outcomes.^24^ However, some limitations exist. First, it was not possible to assess directly the actual difference in FGF-23 in UK Biobank predicted by the genetic risk scores. Instead, we provide evidence of the genetic risk score's validity using data from an independent population where it was shown to predict higher levels of FGF-23. Second, although two *cis*-variants were identified, there was limited power for sensitivity analyses using these important, likely more specific, variants.^32^ Nevertheless, point estimates from analyses using these *cis-*variants were consistent with the results from using the full genetic score (Figure 1). Third, the study was restricted to adults of European ancestry and a general population, meaning results may not be generalizable to other populations. In particular, levels of FGF-23 in patients on maintenance hemodialysis are often two orders of magnitude higher than those in general populations.^12^ It is possible disease associations may differ at extremely high concentrations—for example, if there is a “threshold” effect above which FGF-23 becomes toxic. Such a hypothesis is supported by a recent study's findings in which no association between genetically predicted FGF-23 and heart failure overall, but positive associations emerged among individuals with low genetically predicted eGFR.^45^

The difference between the associations of FGF-23 with outcomes observed from conventional epidemiological studies versus using genetic approaches highlights a key challenge to epidemiologists performing observational studies where it is important to adjust for any degree of kidney disease. Adjusting for kidney function using eGFR is unlikely to fully account for any confounding effect of kidney disease because of a combination of inaccuracy in estimation of kidney function and within-person variability,^46,47^ underadjustment for the effect of CKD duration, and imprecise adjustment for any effects of kidney disease/dysfunction not captured by eGFR.

In summary, our previous systematic review and meta-analysis of conventional observational studies suggested a lack of exposure-response relationship between FGF-23 and risk of a range of diseases and that FGF-23 associations are nonspecific.^12^ We now demonstrate that genetically predicted FGF-23 is not associated with risk of atherosclerotic or nonatherosclerotic cardiovascular diseases. The totality of the evidence suggests that FGF-23 does not have a direct causal role in the development of cardiovascular diseases, and directly targeting FGF-23 is therefore unlikely to represent a clinically meaningful modifiable target to prevent cardiovascular disease. Conventionally observed associations between FGF-23 with cardiovascular disease are likely due to residual confounding.

## Supplementary Material

## Supplemental Material

Supplemental Methods. UK Biobank outcome definitions.

Supplemental Table 1. Thirty-four independent genetic variants for FGF-23 identified in SCALLOP.

Supplemental Table 2. FGF-23 variants associated with other cardiovascular risk factors (*i.e.*, potentially pleiotropic SNPs).

Supplemental Table 3. *cis*-variant associations with logFGF-23 concentration.

Supplemental Table 4. Variance explained and minimum detectable ORs for the 34 SNP FGF-23 genetic score in UK Biobank by outcome.

Supplemental Table 5. Cross-validation of identified variants in four GWAS.

Supplemental Table 6. Summary of populations used for genetic analyses.

Supplemental Table 7. Validation of 34 SNP genetic score using ORIGIN data.

Supplemental Table 8. Associations between genetically predicted FGF-23 with risk of outcomes after excluding four SNPs that are potentially pleiotropic with cardiovascular traits/risk factors.

Supplemental Table 9. Associations between genetically predicted FGF-23 with risk of outcomes after excluding two SNPs that are potentially pleiotropic with eGFR.

Supplemental Table 10. Associations between genetically predicted FGF-23 with risk of outcomes using the eight FGF-23 SNPs associated with FGF-23 levels at *P*<5×10^−8^.

Supplemental Table 11. Associations between genetically predicted FGF-23 with risk of outcomes using only the lead SNP per locus in the genetic instrument.

Supplemental Table 12. Associations between genetically predicted FGF-23 with risk of outcomes excluding SNPs at the *CYP24A1* locus.

Supplemental Table 13. Steiger filtering investigating potential mediating effects of eGFR and BMI.

Supplemental Figure 1. SCALLOP GWAS Q-Q plot and Manhattan plot.

Supplemental Figure 2. Associations between genetically predicted FGF-23 with risk of atherosclerotic cardiovascular outcomes using standard methods to assess the validity of instrumental variable assumptions.

Supplemental Figure 3. Associations between genetically predicted FGF-23 with risk of nonatherosclerotic cardiovascular outcomes using standard methods to assess the validity of instrumental variable assumptions.

Supplemental Figure 4. Associations between genetically predicted FGF-23 with risk of noncardiovascular outcomes using standard methods to assess the validity of instrumental variable assumptions.

Supplemental Figure 5. Associations between genetically predicted FGF-23 with clinical measurements using standard methods to assess the validity of instrumental variable assumptions.

Supplemental Figure 6. Forest plots of effect estimates for individual SNP-FGF-23 associations and associations with key clinical outcomes.

Supplemental Appendix 1. OPCS-4 codes used to define the outcome “other revascularization.”

Supplemental Appendix 2. ICD-10 diagnostic codes used to define hospitalization for infection.
