# Supplementary material for: Fibroblast Growth Factor-23 and Risk of Cardiovascular Diseases: A Mendelian Randomization Study
Source: Clin J Am Soc Nephrol. 2022 Jan 18;18(1):17–27. doi: 10.2215/CJN.05080422 (PMC7614195; doi:10.2215/CJN.05080422)
Supplement: Supplementary file 1 [file cjasn-18-017-s001.pdf]

Fibroblast Growth Factor-23 and Risk of Cardiovascular Diseases: A Mendelian Randomisation Study

Supplemental Materials

Supplemental Methods: UK Biobank outcome definitions

Supplemental Table 1: 34 independent genetic variants for FGF-23 identified in SCALLOP

Supplemental Table 2: FGF-23 variants associated with other cardiovascular risk factors (i.e. potentially pleiotropic SNPs)

Supplemental Table 3: *Cis*-variant associations with logFGF-23 concentration

Supplemental Table 4: Variance explained and minimum detectable ORs for the 34 SNP FGF-23 genetic score in UK Biobank, by outcome

Supplemental Table 5: Cross-validation of identified variants in four GWAS

Supplemental Table 6: Summary of populations used for genetic analyses

Supplemental Table 7: Validation of 34 SNP genetic score using ORIGIN data

Supplemental Table 8: Associations between genetically-predicted FGF-23 with risk of outcomes after excluding four SNPs which are potentially pleiotropic with cardiovascular traits/risk factors

Supplemental Table 9: Associations between genetically-predicted FGF-23 with risk of outcomes after excluding two SNPs which are potentially pleiotropic with eGFR

Supplemental Table 10: Associations between genetically-predicted FGF-23 with risk of outcomes using the 8 FGF-23 SNPs associated with FGF-23 levels at $p<5x10^{-8}$

Supplemental Table 11: Associations between genetically-predicted FGF-23 with risk of outcomes using only the lead SNP per locus in the genetic instrument

Supplemental Table 12: Associations between genetically-predicted FGF-23 with risk of outcomes excluding SNPs at the *CYP24A1* locus

Supplemental Table 13: Steiger filtering investigating potential mediating effects of eGFR and BMI

Supplemental Figure 1: SCALLOP GWAS Q-Q plot and Manhattan plot

Supplemental Figure 2: Associations between genetically-predicted FGF-23 with risk of atherosclerotic cardiovascular outcomes using standard methods to assess validity of instrumental variable assumptions

Supplemental Figure 3: Associations between genetically-predicted FGF-23 with risk of non-atherosclerotic cardiovascular outcomes using standard methods to assess validity of instrumental variable assumptions

Supplemental Figure 4: Associations between genetically-predicted FGF-23 with risk of non-cardiovascular outcomes using standard methods to assess validity of instrumental variable assumptions

Supplemental Figure 5: Associations between genetically-predicted FGF-23 with clinical measurements using standard methods to assess validity of instrumental variable assumptions

Supplemental Figure 6: Forest plots of effect estimates for individual SNP-FGF-23 associations and associations with key clinical outcomes

Supplemental Appendix 1: OPCS-4 codes used to define the outcome “other revascularization”

Supplemental Appendix 2: ICD-10 diagnostic codes used to define hospitalization for infection

Supplemental Methods: UK Biobank outcome definitions

The following outcomes were defined in UK Biobank using hospital admission and mortality data.

Outcome	Definition	Notes
**Any atherosclerotic cardiovascular outcome**		
Non-fatal myocardial infarction	Participant survived 30 days beyond the first day of a hospital episode with an ICD-10 code of I219, I22, I220, I221, I228, I229, I23, I230, I231, I232, I233, I234, I235, I236 or I238 in any diagnostic position	
Coronary revascularization	Participant had a hospital episode with an OPCS-4 code of K49, K50, K75, K40, K41, K42, K43, K44, K45 or K46	Includes coronary bypass, balloon angioplasty and stenting.
Ischaemic stroke (fatal or not)	Participant had a hospital episode with an ICD-10 code of I63 or I64 in any diagnostic position, or died with ICD-10 code I63 or I64 as the underlying cause of death	Includes strokes not specified as hemorrhagic or ischaemic
Coronary death	Participant died with an underlying cause of death ICD-10 code of I20, I21, I22, I23, I24, I250, I251, I252, I256, I258 or I259	Includes ischaemic cardiomyopathy as a cause of death
Other revascularization	Participant had a hospital episode with any of the OPCS-4 procedural codes code listed in Supplemental appendix 1.	Included carotid revascularization, abdominal viscera/renal revascularization, upper and lower limb (distal to the aortic bifurcation, including aorto-femoral bypass) revascularizations, but excluded other aortic procedures and amputations
**Any non-atherosclerotic cardiovascular outcome**		
Hospitalization with heart failure	Participant had a hospital episode with an ICD-10 code of I50, I501, I509, I110, I130, I132, I255, I420, I423, I424, I425, I426, I427, I428, I429, I43, I430, I431, I432, or I438 in any diagnostic position	Includes cardiomyopathies due to nutritional, metabolic, infectious and drug causes
Non-coronary cardiac death	Participant has died, with the underlying cause of death ICD-10 code of I01, I020, I05, I06, I11, II3, I253, I254, I255, I3, I4, I5 or R96	Includes unexplained sudden death
Other vascular death	Participant has died, with an underlying cause of death ICD-10 code in the range I00-I99, but excluding those already defined as a coronary (see above), other cardiac (see above), or stroke death (I60, I61, I63, I64, I69 and I629)	
Hemorrhagic stroke (fatal or not)	Participant had a hospital episode with an ICD-10 code of I60 or I61 in any diagnostic position, or died with an underlying cause of death ICD-10 code of I60 or I61	Includes subarachnoid hemorrhage
**Non-cardiovascular outcomes**		
Any fracture	Participant answered in the affirmative to "Have you fractured a bone in the last 5 years?" at recruitment, or had a hospital episode with a fracture of any bone.	Includes long bones, axial skeleton, hands, feet and skull
Fragility fracture	Participant had a hospital episode with an ICD-10 code of M800, M801, M802, M803, M804, M805, M808, M809, M844, S220, S221, S320, S321, S322, S323, S324, S325, S327, S720, S721, or S722	Includes specified osteoporotic fragility fractures (M80X), fractures of thoracolumbar vertebrae, the pelvic rim and the neck of femur.

Outcome	Definition	Notes
Hospitalization with acute kidney injury	Participant had a hospital episode with ICD-10 code N17 in any diagnostic position	
Hospitalization for infection	Participant had a hospital episode with an ICD-10 code as listed in Supplemental appendix 2	Defined as bacteria or parasitic infection that would usually require antimicrobial treatment. Definitions adapted from https://doi.org/10.1136/bmj.k5092
End-stage kidney disease (treated)	Maintenance dialysis or kidney transplant recipient	
Non cardiovascular death	Participant has died, with the underlying cause of death ICD-10 which is neither in the range I00-I99, nor R96	

Supplemental Table 1: 34 independent genetic variants for FGF-23 identified in SCALLOP

SNP rsID	Marker location	Chr. band	Alleles a1	Alleles a2	Nearest gene	Minor allele frequency	Association with logFGF-23: Effect size (beta)	Standard error of beta	p value	Number of participants with data
rs6706281	2:190452748	2q32.2	A	C	*SLC40A1*	0.29	-0.1057	0.0121	2.43E-18	18239
rs2870308	20:52727953	20q13.2	A	C	*BCAS1*	0.27	-0.0977	0.0125	4.70E-15	18239
rs687289	9:136137106	9q34.2	A	G	*ABO*	0.37	0.0776	0.0118	5.45E-11	15273
rs11748297	5:176800361	5q35.3	A	G	*RGS14*	0.29	-0.0709	0.0113	3.57E-10	19195
rs6489536 §	*12:4491909*	*12p13.32*	*C*	G	*FGF23*	*0.33*	*0.0702*	*0.0122*	*7.77E-09*	*15802*
rs4744712	9:71434707	9q21.11	A	C	*PIP5K1B*	0.39	-0.0596	0.0104	1.08E-08	19195
rs34551523	5:146629145	5q32	A	G	*STK32A*	0.03	0.2015	0.0358	1.82E-08	15801
rs6561643	13:33509079	13q13.1	A	T	*KL*	0.37	-0.0651	0.0118	3.18E-08	18239
rs16988687	20:42704488	20q13.12	T	C	*TOX2*	0.15	-0.0785	0.0146	7.44E-08	19195
rs9372822	6:125928290	6q22.31	T	C	*RP11-624M8.1*	0.35	-0.0591	0.0112	1.17E-07	19195
rs74461633	6:166587846	6q27	T	C	*SNORD45*	0.04	0.1663	0.0317	1.54E-07	15801
rs13038432	20:52787302	20q13.2	A	G	*CYP24A1*	0.08	0.1107	0.0214	2.30E-07	18239
rs7955866 §	*12:4479549*	*12p13.32*	*A*	G	*FGF23*	*0.12*	*-0.0915*	*0.0178*	*2.65E-07*	*15801*
rs150865155	12:117088921	12q24.22	A	G		0.01	-0.3124	0.0626	5.90E-07	13990
rs35827013	2:155016134	2q24.1	A	G	*GALNT13*	0.27	0.0619	0.0124	6.33E-07	19195
rs192539378	2:190581687	2q32.2	A	G	*ANKAR*	0.05	-0.1394	0.028	6.59E-07	13932
rs1570669	20:52774427	20q13.2	A	G	*CYP24A1*	0.34	-0.0548	0.0112	9.19E-07	18239
rs186557449	3:20739233	3p24.3	T	C		0.02	-0.2606	0.0531	9.20E-07	14501
rs67833371	18:8702913	18p11.22	A	T		0.37	0.0647	0.0132	1.01E-06	12976
rs147720247	16:65073369	16q21	T	G	*CDH11*	0.04	0.1493	0.0311	1.59E-06	17326
rs11035939	11:40600846	11p12	A	G	*LRRC4C*	0.28	-0.0599	0.0125	1.77E-06	15801
rs189972262	4:12448579	4p15.33	T	C		0.02	0.3595	0.0754	1.86E-06	9788
rs2720020	3:163629696	3q26.1	T	G		0.21	0.0651	0.0137	1.86E-06	19195
rs147321547	19:51883001	19q13.41	T	C	*LIM2*	0.19	-0.0705	0.0148	2.03E-06	15801
rs117612483	13:111392745	13q34	T	G	*ING1*	0.02	0.2503	0.0533	2.63E-06	14624
rs290403	20:52711514	20q13.2	A	G	*CYP24A1*	0.47	0.0524	0.0112	2.93E-06	18239
rs78450448	15:53626754	15q21.3	T	C	*WDR72*	0.02	0.202	0.0432	2.96E-06	15457
rs117989952	8:4020568	8p23.2	A	G	*CSMD1*	0.01	0.5645	0.1209	3.05E-06	8067

SNP rsID	Marker location	Chr. band	Alleles a1	a2	Nearest gene	Minor allele frequency	Association with logFGF-23 Effect size (beta)	Standard error of beta	p value	Number of participants with data
rs75357988	5:161568285	5q34	T	C	*GABRG2*	0.02	-0.225	0.0483	3.19E-06	14390
rs11542063	15:45353365	15q21.1	A	G	*SORD*	0.02	0.2488	0.0536	3.45E-06	14624
rs61855139	10:70223133	10q21.3	A	G	*DNA2*	0.10	-0.0888	0.0193	4.33E-06	17326
rs79146532	17:34934676	17q12	A	G	*GGNBP2*	0.03	-0.1618	0.0353	4.41E-06	19195
rs9695235	9:119138128	9q33.1	T	C	*PAPPA*	0.20	0.0611	0.0134	4.77E-06	19195
rs11217709	11:120029788	11q23.3	T	C	*TRIM29*	0.04	-0.1539	0.0337	4.83E-06	14845

FGF-23, Fibroblast growth factor 23; SNP, single nucleotide polymorphism. § denotes the 2 *cis*-variants within 100kb of the FGF-23 transcription start site. Marker locations uses GRCh37 co-ordinates. Allele1 is the effect allele. Effects are measured in standard deviations of $\log_2$ transformed FGF-23.

Supplemental Table 2: FGF-23 variants associated with other cardiovascular trait/risk factors (i.e. potentially pleiotropic SNPs)

SNP rsID	Alleles		Trait/ risk factor	Data source	Ancestry	Year	Association with trait/risk factor				no. with data	no. of studies	unit of analysis
	a1	a2					Effect size (beta)	Standard error of beta	p value	Direction			
rs687289	G	A	Low density lipoprotein	PMID 24097068	European	2013	-0.0403	0.004	1.33E-24	-	172684	60	IVNT
	G	A	Low density lipoprotein	PMID 20686565	Mixed	2010	-0.0433	0.005	5.74E-16	-	95454	46	z score
	G	A	Total cholesterol	PMID 24097068	European	2013	-0.0403	0.004	2.14E-26	-	186901	60	IVNT
	G	A	Total cholesterol	PMID 20686565	Mixed	2010	-0.0408	0.0052	6.09E-15	-	100184	46	z score
	G	A	Diastolic BP	UK Biobank	European	2017	0.01664	0.002643	3.09E-10	+	317756	1	IVNT
	G	A	Doctor diagnosed : hypertension	UK Biobank	European	2017	0.006404	0.001161	3.49E-08	+	336683	1	Risk difference
rs6561643	A	T	Type 2 DM	PMID 28566273	European	2017	0.04	0.014	0.004	+	159208	18	logOR
rs79146532	A	G	Tobacco smoking: (occasional)	UK Biobank	European	2017	-0.00536	0.001751	0.002	-	83133	1	Risk difference
rs4744712	A	C	Glomerular filtration rate	PMID 26831199	European	2016	0.0071		4.00E-15	+	133720	1	ml/min/1.73 m^2

FGF-23, Fibroblast growth factor 23; SNP, single nucleotide polymorphism; BP, blood pressure; DM, diabetes mellitus; PMID, Pubmed ID; IVNT= inverse normally ranked phenotype; OR=odds ratio. Associations with cardiovascular traits/risk factors for the 34x FGF-23 SNPs (and variants in linkage disequilibrium [r^2>0.8; 1000 Genomes Phase 3 release]) identified from published data from PhenoScanner (http://www.phenoscanner.medschl.cam.ac.uk/about/)

Supplemental Table 3: *Cis*-variant associations with logFGF-23 concentration

SNP rsID	Increase in logFGF-23 per effect allele (standard error)	p values	
		unadjusted	conditioned on other *cis*-variant
rs6489536	0.0702 (0.012)	$7.8x10^{-9}$	$5.4x10^{-6}$
rs7955866	-0.0915 (0.0178)	$2.7x10^{-7}$	$1.8x10^{-4}$

FGF-23, Fibroblast growth factor 23; SNP, single nucleotide polymorphism

Supplemental Table 4: Variance explained and minimum detectable ORs for the 34 SNP FGF-23 genetic score in UK Biobank, by outcome

	Proportion of participants with outcome	Proportion of variance of logFGF-23 explained	Minimal detectable OR per 1-SD higher logFGF-23 with 80% power, 2p=0.05
**Atherosclerotic cardiovascular event**			
Non-fatal myocardial infarction	0.029		1.12
Ischemic stroke	0.018		1.15
Coronary revascularization	0.043		1.10
Other revascularization	0.011	0.063	1.19
Coronary death	0.007		1.24
**Any atherosclerotic cardiovascular event**	**0.078**		**1.08**
**Non-atherosclerotic cardiovascular outcomes**			
Hospitalization with heart failure	0.030		1.12
Hemorrhagic stroke	0.005		1.28
Non-coronary cardiac death	0.002	0.063	1.44
Other vascular death	0.002		1.44
**Any non-atherosclerotic cardiovascular event**	**0.037**		**1.11**
**Non-cardiovascular outcomes**			
Any fracture	0.15		1.06
Fragility fracture	0.020		1.14
Hospitalization for infection	0.114		1.07
Hospitalization with acute kidney injury	0.034	0.063	1.12
End-stage kidney disease (treated)	0.002		1.36
Non-cardiovascular death	0.054		1.09

FGF-23, Fibroblast growth factor 23; OR, odds ratio; SNP, single nucleotide polymorphism; SD, standard deviation

Supplemental Table 5: Cross-validation of identified variants in four GWAS

A: Top variants from Robinson-Cohen GWAS[21]

Variant	p-value in Robinson-Cohen GWAS	p-value in SCALLOP GWAS	Concordant effect direction?
rs11741640	$1.6x10^{-16}$	$6.4x10^{-10}$	Yes
rs2769071	$6.1x10^{-17}$	$1.3x10^{-10}$	Yes
rs9925837	$5.1x10^{-9}$	NR	NA
rs17216707	$3.0x10^{-24}$	NR	NA
rs17479566	$2.0x10^{-9}$	NR	NA

B: *cis*-variants from SCALLOP GWAS

Variant	p-value in SCALLOP GWAS	p-value in Scottish GWAS	Concordant direction with SCALLOP?	p-value in Icelandic GWAS	Concordant direction with SCALLOP?
rs6489536	$7.8x10^{-9}$	$1.3x10^{-2}$	Yes	$3.9x10^{-6}$	Yes
rs7955866	$2.7 x 10^{-7}$	$1.8x10^{-2}$	Yes	$2.3x10^{-1}$	Yes

Supplemental Table 6: Summary of populations used for genetic analyses

	SCALLOP	ORIGIN	UK Biobank
	Ancestry: European	Ancestry: European (44%), Native Latin (50%), African ancestry (6%)	Ancestry: European subset only used
**Variant**	**Minor Allele Frequency**	**Minor Allele Frequency**	**Minor Allele Frequency**
rs11035939	0.283	0.321	0.283
rs11217709	0.041	0.031	0.040
rs11542063	0.023	0.007	0.020
rs11748297	0.290	0.248	0.265
rs117612483	0.018	0.007	0.011
rs117989952	0.007	0.005	0.005
rs13038432	0.083	0.058	0.076
rs147321547	0.189	0.145	0.191
rs147720247	0.040	0.035	0.037
rs150865155	0.011	0.006	0.010
rs1570669	0.344	0.363	0.351
rs16988687	0.150	0.112	0.154
rs186557449	0.018	0.008	0.012
rs189972262	0.022	0.004	0.003
rs192539378	0.048	0.032	0.045
rs2720020	0.205	0.149	0.187
rs2870308	0.268	0.328	0.260
rs290403	0.471	0.397	0.444
rs34551523	0.029	0.024	0.029
rs35827013	0.267	0.221	0.257
rs4744712	0.387	0.362	0.397
rs61855139	0.102	0.115	0.086
rs6489536	0.326	0.320	0.340
rs6561643	0.369	0.373	0.356
rs6706281	0.289	0.278	0.270
rs67833371	0.374	0.361	0.376
rs687289	0.373	0.342	0.324
rs74461633	0.036	0.025	0.026
rs75357988	0.025	0.015	0.010
rs78450448	0.019	0.014	0.017
rs79146532	0.028	0.011	0.029
rs7955866	0.119	0.146	0.117
rs9372822	0.346	0.370	0.368
rs9695235	0.196	0.200	0.184

Supplemental Table 7: Validation of 34 SNP genetic score using ORIGIN data

Range of FGF23 concentrations (pg/mL)	No. of participants	OR (95% CI) of being in this category or a higher category, per 1 unit higher genetic score	p value
<49	2575	--	
[49 – 60]	430	1.05 (0.99 – 1.12)	
[60 – 80]	485	1.07 (1.00 – 1.15)	
[80 – 120]	389	1.10 (1.02 – 1.18)	
[120 – 4500]	511	1.13 (1.03 – 1.24)	
**OVERALL**	**4390**	**1.07 (1.00 – 1.13)**	**0.036**

FGF-23, Fibroblast growth factor 23; SNP= single nucleotide polymorphism; OR (95% CI), odds ratio (95% confidence interval). Analyses use ordinal regression adjusted for age, sex and ethnicity. Ordinal regression was used due to the high proportion of participants with values of FGF-23 below the lower limit of detection for the assay.

Supplemental Table 8: Associations between genetically-predicted FGF-23 with risk of outcomes after excluding three SNPs which are potentially pleiotropic with cardiovascular traits/risk factors

Outcome	Number of outcomes	OR per 1-SD higher genetically-predicted logFGF-23 (95% CI)	p-value (Bonferroni corrected)
**Atherosclerotic cardiovascular outcomes**			
Non-fatal myocardial infarction	9,677	1.06 (0.98 – 1.16)	0.99
Ischemic stroke	5,992	1.01 (0.91 – 1.13)	0.99
Coronary revascularization	14,646	1.03 (0.96 – 1.10)	0.98
Other revascularization	3,782	0.97 (0.85 – 1.12)	0.99
Coronary death	2,258	1.03 (0.96 – 1.10)	0.99
**Any atherosclerotic cardiovascular event**	**26,266**	**1.02 (0.97 – 1.08)**	**0.99**
**Non-atherosclerotic cardiovascular outcomes**			
Hospitalization with heart failure	10,177	1.00 (0.92 – 1.09)	0.99
Hemorrhagic stroke	1,745	1.07 (0.88 – 1.30)	0.99
Non-coronary cardiac death	689	0.95 (0.69 – 1.30)	0.99
Other vascular death	617	0.98 (0.70 – 1.36)	0.99
**Any non-atherosclerotic cardiovascular event**	**12,652**	**1.01 (0.94 – 1.09)**	**0.99**
**Non-cardiovascular outcomes**			
Any fracture	51,166	1.00 (0.96 – 1.04)	0.99
Fragility fracture	6,624	1.04 (0.94 – 1.16)	0.99
Hospitalization for infection	38,613	1.01 (0.97 – 1.06)	0.99
Hospitalization with acute kidney injury	11,569	0.96 (0.89 – 1.04)	0.99
End-stage kidney disease (treated)	774	1.07 (0.79 – 1.43)	0.99
Non-vascular death	17,196	1.01 (0.94 – 1.08)	0.99
**Clinical measurement (units)**	**Number with measurement**	**Effect of 1-SD higher genetically predicted logFGF-23 (95% CI)**	
Android bone mass (grams)	3,695	1.48 (-0.03,2.98)	0.51
Gynoid bone mass (grams)	3,695	9.42 (3.46,15.38)	0.03
Lumbar vertebral bone mineral density (g/cm^3)	3,679	0.013 (0.001,0.039)	0.42
Femoral neck bone mineral density (g/cm^3)	3,704	0.009 (0.002,0.039)	0.31
Carotid intimal thickness (maximum; μm)	31,641	0.4 (-5.8,6.6)	0.99
Carotid intimal thickness (mean; μm)	31,641	-0.8 (-6.0,4.4)	0.99
Left ventricular mass index (g/m^2)	18,710	0.29 (-0.10,0.69)	0.87

FGF-23, Fibroblast growth factor 23; SNP= single nucleotide polymorphism; OR (95% CI), odds ratio (95% confidence interval); SD, standard deviation. Excluded SNP rsID's: rs687289, rs6561643, rs79146532 and rs4744712

Supplemental Table 9: Associations between genetically-predicted FGF-23 with risk of outcomes after excluding two SNPs which are potentially pleiotropic with eGFR

Outcome	Number of outcomes	OR per 1-SD higher genetically-predicted logFGF-23 (95% CI)	p-value (Bonferroni corrected)
**Atherosclerotic cardiovascular outcomes**			
Non-fatal myocardial infarction	9,677	1.08 (0.99 – 1.17)	0.72
Ischemic stroke	5,992	1.05 (0.95 – 1.17)	0.99
Coronary revascularization	14,646	1.02 (0.95 – 1.09)	0.99
Other revascularization	3,782	1.02 (0.89 – 1.17)	0.99
Coronary death	2,258	1.15 (0.97 – 1.37)	0.80
**Any atherosclerotic cardiovascular event**	**26,266**	**1.04 (0.98 – 1.10)**	**0.91**
**Non-atherosclerotic cardiovascular outcomes**			
Hospitalization with heart failure	10,177	1.02 (0.94 – 1.10)	0.99
Hemorrhagic stroke	1,745	1.08 (0.89 – 1.32)	0.99
Non-coronary cardiac death	689	0.96 (0.70 – 1.31)	0.99
Other vascular death	617	0.92 (0.66 – 1.28)	0.99
**Any non-atherosclerotic cardiovascular event**	**12,652**	**1.02 (0.95 – 1.10)**	**0.99**
**Non-cardiovascular outcomes**			
Any fracture	51,166	1.00 (0.96 – 1.04)	0.99
Fragility fracture	6,624	1.06 (0.96 – 1.17)	0.98
Hospitalization for infection	38,613	1.01 (0.96 – 1.05)	0.99
Hospitalization with acute kidney injury	11,569	0.95 (0.88 – 1.03)	0.96
End-stage kidney disease (treated)	774	1.02 (0.76 – 1.37)	0.99
Non-cardiovascular death	17,196	1.00 (0.94 – 1.06)	0.99
**Clinical measurement (units)**	**Number with measurement**	**Effect of 1-SD higher genetically predicted logFGF-23 (95% CI)**	
Android bone mass (g)	3,695	1.41 (-0.09,2.91)	0.59
Gynoid bone mass (g)	3,695	9.76 (3.81,15.71)	0.02
Lumbar vertebral bone mineral density (g/cm^3)	3,679	0.027 (0.001,0.053)	0.43
Femoral neck bone mineral density (g/cm^3)	3,704	0.021 (0.002,0.039)	0.32
Carotid intimal thickness (maximum; μm)	31,641	1.4 (-4.8,7.6)	0.99
Carotid intimal thickness (mean; μm)	31,641	0.2 (-5.0,5.3)	0.99
Left ventricular mass index (g/m^2)	18,710	0.40 (0.01,0.79)	0.46

FGF-23, Fibroblast growth factor 23; SNP= single nucleotide polymorphism; OR (95% CI), odds ratio (95% confidence interval); SD, standard deviation. Excluded SNPs are rs4744712 and rs2870308

Supplemental Table 10: Associations between genetically-predicted FGF-23 with risk of outcomes using the 8 FGF-23 SNPs associated with FGF-23 levels at $p<5x10^{-8}$

Outcome	Number of outcomes	OR per 1-SD higher genetically-predicted logFGF-23 (95% CI)	p-value (Bonferroni corrected)
**Atherosclerotic cardiovascular outcomes**			
Non-fatal myocardial infarction	9,677	1.15 (1.00 – 1.32)	0.72
Ischemic stroke	5,992	0.98 (0.82 – 1.18)	0.99
Coronary revascularization	14,646	1.07 (0.95 – 1.20)	0.99
Other revascularization	3,782	1.04 (0.83 – 1.30)	0.73
Coronary death	2,258	1.08 (0.81 – 1.44)	0.99
**Any atherosclerotic cardiovascular event**	**26,266**	**1.06 (0.97 – 1.16)**	**0.99**
**Non-atherosclerotic cardiovascular outcomes**			
Hospitalization with heart failure	10,177	1.01 (0.88 – 1.16)	0.99
Hemorrhagic stroke	1,745	1.00 (0.72 – 1.39)	0.99
Non-coronary cardiac death	689	0.68 (0.40 – 1.15)	0.97
Other vascular death	617	0.93 (0.54 – 1.62)	0.99
**Any non-atherosclerotic cardiovascular event**	**12,652**	**0.98 (0.87 – 1.12)**	**0.99**
**Non-cardiovascular outcomes**			
Any fracture	51,166	1.03 (0.97 – 1.10)	0.99
Fragility fracture	6,624	1.29 (1.09 – 1.53)	0.07
Hospitalization for infection	38,613	1.07 (0.99 – 1.15)	0.84
Hospitalization with acute kidney injury	11,569	0.97 (0.85 – 1.10)	0.99
End-stage kidney disease (treated)	774	1.02 (0.62 – 1.67)	0.99
Non-cardiovascular death	17,196	1.02 (0.92 – 1.14)	0.99
**Clinical measurement (units)**	**Number with measurement**	**Effect of 1-SD higher genetically predicted logFGF-23 (95% CI)**	
Android bone mass (g)	3,695	-0.31 (-2.83,2.21)	0.99
Gynoid bone mass (g)	3,695	4.01 (-5.97,14.04)	0.99
Lumbar vertebral bone mineral density (g/cm^3)	3,679	0.005 (-0.039,0.048)	0.99
Femoral neck bone mineral density (g/cm^3)	3,704	0.005 (-0.026,0.036)	0.99
Carotid intimal thickness (maximum; μm)	31,641	0.3 (-10.1,10.7)	0.99
Carotid intimal thickness (mean; μm)	31,641	0.4 (-8.3,9.1)	0.99
Left ventricular mass index (g/m^2)	18,710	0.99 (0.33,1.65)	0.07

FGF-23, Fibroblast growth factor 23. The 8 contributory SNPs are indicated in Supplemental Table 1

Supplemental Table 11: Associations between genetically-predicted FGF-23 with risk of outcomes using only the lead SNP per locus in the genetic instrument

Outcome	Number of outcomes	OR per 1-SD higher genetically-predicted logFGF-23 (95% CI)	p-value (Bonferroni corrected)
**Atherosclerotic cardiovascular outcomes**			
Non-fatal myocardial infarction	9,677	0.96 (0.87 – 1.06)	0.99
Ischemic stroke	5,992	0.95 (0.84 – 1.07)	0.99
Coronary revascularization	14,646	1.01 (0.93 – 1.09)	0.99
Other revascularization	3,782	0.95 (0.82 – 1.11)	0.99
Coronary death	2,258	0.90 (0.74 – 1.10)	0.99
**Any atherosclerotic cardiovascular event**	**26,266**	**0.98 (0.92 – 1.04)**	**0.99**
**Non-atherosclerotic cardiovascular outcomes**			
Hospitalization with heart failure	10,177	0.93 (0.85 – 1.02)	0.87
Haemorrhagic stroke	1,745	1.06 (0.85 – 1.33)	0.99
Non-coronary cardiac death	689	1.09 (0.76 – 1.56)	0.99
Other vascular death	617	0.74 (0.51 – 1.07)	0.78
**Any non-atherosclerotic cardiovascular event**	**12,652**	**0.95 (0.87 – 1.03)**	**0.96**
**Non-cardiovascular outcomes**			
Any fracture	51,166	1.03 (0.98 – 1.08)	0.94
Fragility fracture	6,624	1.05 (0.93 – 1.18)	0.99
Hospitalization for infection	38,613	1.00 (0.95 – 1.05)	0.99
Hospitalization with acute kidney injury	11,569	0.94 (0.86 – 1.03)	0.95
End-stage kidney disease (treated)	774	1.04 (0.74 – 1.46)	0.99
Non-cardiovascular death	17,196	0.97 (0.90 – 1.05)	0.99
**Clinical measurement (units)**	**Number with measurement**	**Effect of 1-SD higher genetically predicted logFGF-23 (95% CI)**	
Android bone mass (g)	3,695	0.66 (-1.05,2.38)	0.99
Gynoid bone mass (g)	3,695	3.48 (-3.35,10.30)	0.99
Lumbar vertebral bone mineral density (g/cm^3)	3,679	0.013 (-0.017,0.043)	0.99
Femoral neck bone mineral density (g/cm^3)	3,704	-0.002 (-0.023,0.019)	0.94
Carotid intimal thickness (maximum; μm)	31,641	3.8 (-3.4,10.9)	0.99
Carotid intimal thickness (mean; μm)	31,641	3.5 (-2.4.,9.5)	0.98
Left ventricular mass index (g/m^2)	18,710	0.45 (-0.01,0.91)	0.51

FGF-23, Fibroblast growth factor 23. The SNPs from the 34 SNP score excluded from this analysis are: rs7955866, rs11542063, rs192539378, rs16988687, rs290403, rs1570669, rs13038432, rs34551523, rs75357988, rs9695235.

Supplemental Table 12: Associations between genetically-predicted FGF-23 with risk of outcomes excluding SNPs at the *CYP24A1* locus

Outcome	Number of outcomes	OR per 1-SD higher genetically-predicted logFGF-23 (95% CI)	p-value (Bonferroni corrected)
**Atherosclerotic cardiovascular outcomes**			
Non-fatal myocardial infarction	9,677	1.05 (0.96 – 1.15)	0.96
Ischemic stroke	5,992	1.04 (0.93 – 1.16)	0.99
Coronary revascularization	14,646	1.01 (0.93 – 1.08)	0.99
Other revascularization	3,782	0.99 (0.86 – 1.14)	0.99
Coronary death	2,258	1.13 (0.94 – 1.35)	0.91
**Any atherosclerotic cardiovascular event**	**26,266**	**1.02 (0.96 – 1.08)**	**0.99**
**Non-atherosclerotic cardiovascular outcomes**			
Hospitalization with heart failure	10,177	1.02 (0.93 – 1.11)	0.99
Haemorrhagic stroke	1,745	1.11 (0.90 – 1.36)	0.98
Non-coronary cardiac death	689	0.99 (0.72 – 1.37)	0.99
Other vascular death	617	0.89 (0.63 – 1.25)	0.99
**Any non-atherosclerotic cardiovascular event**	**12,652**	**1.03 (0.95 – 1.11)**	**0.99**
**Non-cardiovascular outcomes**			
Any fracture	51,166	1.00 (0.96 – 1.04)	0.99
Fragility fracture	6,624	1.05 (0.95 – 1.17)	0.98
Hospitalization for infection	38,613	1.01 (0.96 – 1.05)	0.99
Hospitalization with acute kidney injury	11,569	0.96 (0.86 – 1.04)	0.97
End-stage kidney disease (treated)	774	1.00 (0.74 – 1.35)	0.99
Non-cardiovascular death	17,196	0.99 (0.93 – 1.06)	0.99
**Clinical measurement (units)**	**Number with measurement**	**Effect of 1-SD higher genetically predicted logFGF-23 (95% CI)**	
Android bone mass (g)	3,695	1.18 (-0.36,2.73)	0.83
Gynoid bone mass (g)	3,695	7.71 (1.57,13.85)	0.22
Lumbar vertebral bone mineral density (g/cm^3)	3,679	0.022 (-0.004,0.049)	0.75
Femoral neck bone mineral density (g/cm^3)	3,704	0.012 (-0.007,0.031)	0.94
Carotid intimal thickness (maximum; μm)	31,641	1.24 (-5.2,7.7)	0.99
Carotid intimal thickness (mean; μm)	31,641	0.6 (-4.8,5.9)	0.99
Left ventricular mass index (g/m^2)	18,710	0.35 (-0.06,0.76)	0.74

FGF-23, Fibroblast growth factor 23. The excluded SNPs are indicated in Supplemental Table 1

Supplemental Table 13: Steiger filtering investigating potential mediating effects of eGFR and BMI

Variant	Proportion variance explained of FGF-23	Proportion of variance explained of eGFR (CKDGen)	Proportion of variance explained of BMI (GIANT)
**rs11035939**	1.5E-03	1.8E-06	7.46E-06
**rs11217709**	1.4E-03	6.7E-06	
**rs11748297**	2.0E-03	2.9E-04	
**rs117612483**	1.5E-03	5.0E-08	
**rs13038432**	1.5E-03	1.0E-04	4.08E-06
**rs147321547**	1.4E-03	2.4E-06	
**rs150865155**	1.8E-03	1.5E-07	
**rs1570669**	1.3E-03	9.4E-05	2.11E-07
**rs16988687**	1.5E-03	1.0E-06	6.98E07
**rs186557449**	1.7E-03	1.3E-06	
**rs192539378**	1.8E-03	1.6E-05	
**rs2720020**	1.2E-03	4.6E-07	
**rs2870308**	3.3E-03	1.3E-04	
**rs290403**	1.2E-03	6.7E-05	1.23E-06
**rs34551523**	2.0E-03	1.5E-07	
**rs35827013**	1.3E-03	1.8E-06	
**rs4744712**	1.7E-03	2.7E-04	8.31E-06
**rs61855139**	1.2E-03	3.8E-05	
**rs6489536**	2.1E-03	2.0E-05	3.89E-08
**rs6561643**	1.7E-03	1.2E-07	6.61E-06
**rs6706281**	4.2E-03	4.0E-06	3.47E-08
**rs687289**	2.8E-03	3.5E-05	2.68E-06
**rs74461633**	1.7E-03	5.1E-06	
**rs75357988**	1.5E-03	4.2E-09	
**rs78450448**	1.4E-03	4.0E-06	
**rs79146532**	1.1E-03	6.4E-06	
**rs7955866**	1.7E-03	5.1E-06	5.57E-06
**rs9372822**	1.4E-03	2.9E-06	1.39E-06
**rs9695235**	1.1E-03	1.9E-05	1.25E-05

Supplemental Figure 1: SCALLOP GWAS Q-Q plot and Manhattan plot

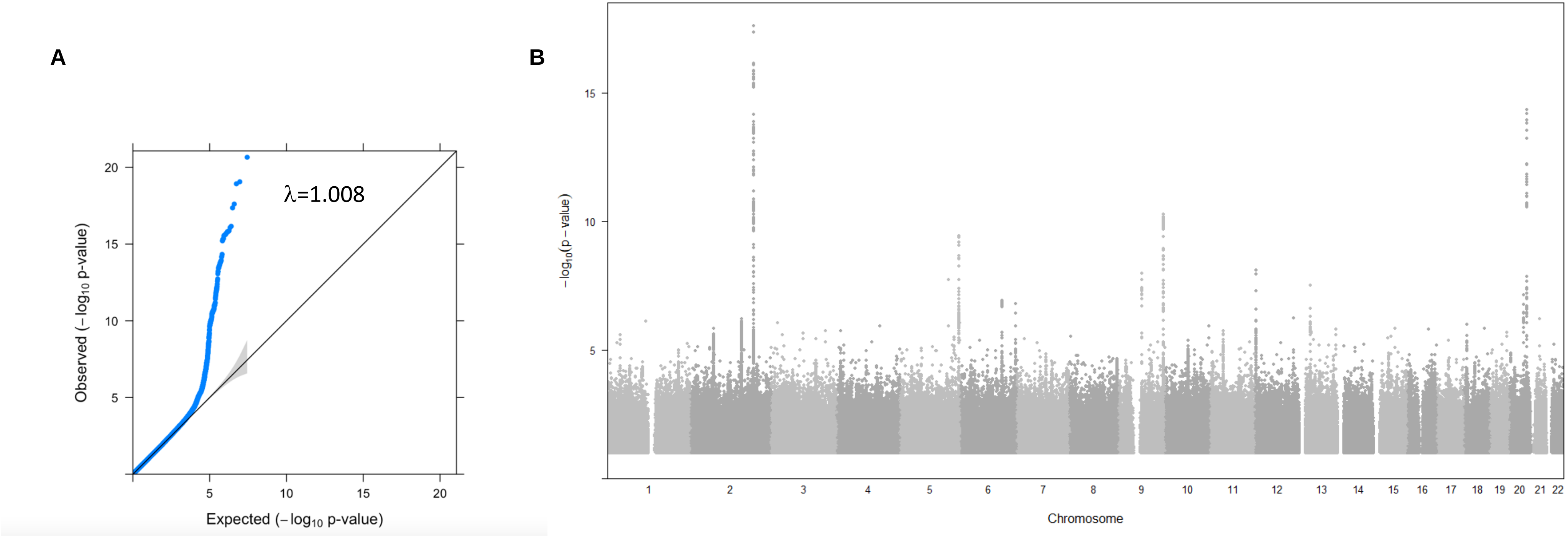

Chi-square Q-Q plot (A) and Manhattan plot (B) of the FGF-23 GWAS meta-analysis in SCALLOP. λ represents the genomic inflation factor.

Supplemental Figure 2: Associations between genetically−predicted FGF−23 with risk of atherosclerotic cardiovascular outcomes using standard methods to assess validity of instrumental variable assumptions (sensitivity analysis)

Outcome	Method	OR (95% CI) per 1−SD higher genetically−predicted logFGF−23	p−value	Intercept	SE intercept	p−value intercept
Non−fatal myocardial infarction	IVW	1.06 (0.98, 1.16)	0.167			
	MR−Egger	0.98 (0.81, 1.19)	0.844	0.008	0.009	0.372
	Weighted median	1.07 (0.95, 1.21)	0.265			
	Outlier corrected IVW	1.06 (0.98, 1.16)	0.167			
Coronary revascularisation	IVW	1.01 (0.94, 1.08)	0.818			
	MR−Egger	0.94 (0.81, 1.11)	0.489	0.006	0.007	0.379
	Weighted median	1.02 (0.92, 1.13)	0.697			
	Outlier corrected IVW	1.01 (0.94, 1.08)	0.818			
Coronary death	IVW	1.15 (0.97, 1.37)	0.116			
	MR−Egger	1.10 (0.74, 1.64)	0.636	0.004	0.018	0.817
	Weighted median	1.11 (0.87, 1.42)	0.414			
	Outlier corrected IVW	1.15 (0.97, 1.37)	0.116			
Ischaemic stroke	IVW	1.04 (0.94, 1.16)	0.439			
	MR−Egger	0.92 (0.72, 1.18)	0.521	0.012	0.011	0.276
	Weighted median	1.05 (0.90, 1.22)	0.529			
	Outlier corrected IVW	1.04 (0.94, 1.16)	0.439			
Other revascularisation	IVW	0.98 (0.86, 1.12)	0.805			
	MR−Egger	0.91 (0.67, 1.24)	0.554	0.007	0.014	0.590
	Weighted median	0.90 (0.74, 1.09)	0.288			
	Outlier corrected IVW	0.98 (0.86, 1.12)	0.805			
**Any atherosclerotic cardiovascular event**	**IVW**	**1.02 (0.97, 1.08)**	**0.397**			
	**MR−Egger**	**0.91 (0.80, 1.03)**	**0.142**	**0.011**	**0.006**	**0.041**
	**Weighted median**	**1.01 (0.93, 1.10)**	**0.776**			
	**Outlier corrected IVW**	**1.02 (0.97, 1.08)**	**0.397**			

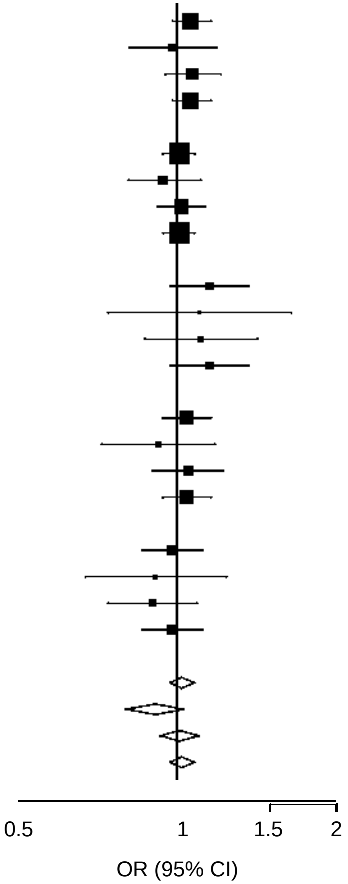

OR (95% CI), odds ratio (95% confidence interval); SD, standard deviation. SE, standard error; IVW=inverse variance weighted. Outlier corrected IVW used the modified Q statistic.

Supplemental Figure 3: Associations between genetically−predicted FGF−23 with risk of non−atherosclerotic cardiovascular outcomes using standard methods to assess validity of instrumental variable assumptions (sensitivity analysis)

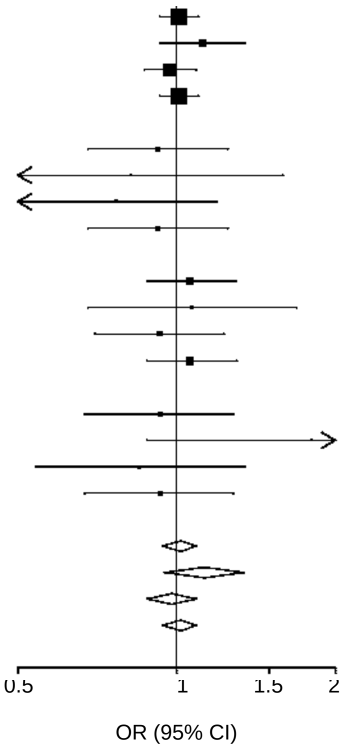

Outcome	Method	OR (95% CI) per 1−SD higher genetically−predicted logFGF−23	p−value	Intercept	SE intercept	p−value intercept
Hospitalisation for heart failure	IVW	1.01 (0.93, 1.10)	0.762			
	MR−Egger	1.12 (0.93, 1.35)	0.242	−0.010	0.008	0.249
	Weighted median	0.97 (0.87, 1.09)	0.656			
	Outlier corrected IVW	1.01 (0.93, 1.10)	0.762			
Other (non−coronary) cardiac death	IVW	0.92 (0.68, 1.25)	0.601			
	MR−Egger	0.82 (0.43, 1.59)	0.562	0.012	0.030	0.702
	Weighted median	0.77 (0.49, 1.19)	0.242			
	Outlier corrected IVW	0.92 (0.68, 1.25)	0.601			
Haemorrhagic stroke	IVW	1.06 (0.88, 1.30)	0.528			
	MR−Egger	1.07 (0.68, 1.69)	0.762	−0.001	0.020	0.973
	Weighted median	0.93 (0.70, 1.23)	0.599			
	Outlier corrected IVW	1.06 (0.88, 1.30)	0.528			
Other vascular death	IVW	0.93 (0.67, 1.28)	0.641			
	MR−Egger	1.80 (0.88, 3.71)	0.110	−0.066	0.033	0.043
	Weighted median	0.85 (0.54, 1.35)	0.493			
	Outlier corrected IVW	0.93 (0.67, 1.28)	0.641			
**Any non−atherosclerotic cardiovascular event**	**IVW**	**1.02 (0.94, 1.09)**	**0.685**			
	**MR−Egger**	**1.13 (0.95, 1.34)**	**0.155**	**−0.011**	**0.008**	**0.166**
	**Weighted median**	**0.98 (0.88, 1.09)**	**0.768**			
	**Outlier corrected IVW**	**1.02 (0.94, 1.09)**	**0.685**			

OR (95% CI), odds ratio (95% confidence interval); SD, standard deviation. SE, standard error; IVW=inverse variance weighted. Outlier corrected IVW used the modified Q statistic.

Supplemental Figure 4: Associations between genetically−predicted FGF−23 with risk of non−cardiovascular outcomes using standard methods to assess validity of instrumental variable assumptions (sensitivity analysis)

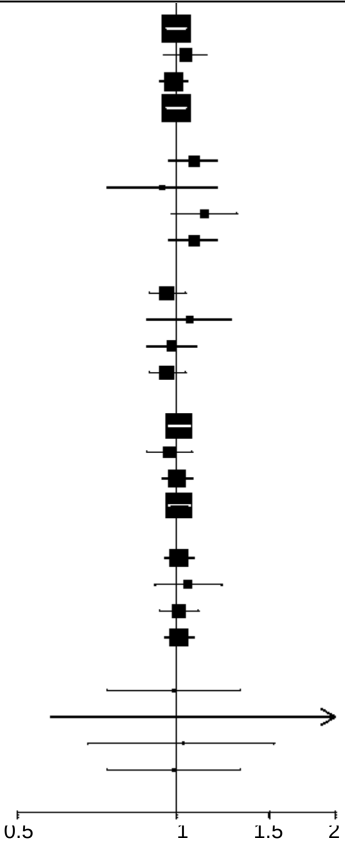

Outcome	Method	OR (95% CI) per 1−SD higher genetically−predicted logFGF−23	p−value	Intercept	SE intercept	p−value intercept
Any fracture	IVW	1.00 (0.96, 1.04)	0.928			
	MR−Egger	1.04 (0.95, 1.14)	0.407	−0.004	0.004	0.381
	Weighted median	0.99 (0.93, 1.05)	0.733			
	Outlier corrected IVW	1.00 (0.96, 1.04)	0.928			
Fragility fracture	IVW	1.08 (0.97, 1.19)	0.162			
	MR−Egger	0.94 (0.74, 1.19)	0.607	0.013	0.010	0.215
	Weighted median	1.13 (0.98, 1.30)	0.104			
	Outlier corrected IVW	1.08 (0.97, 1.19)	0.162			
Hospitalisation with acute kidney injury	IVW	0.96 (0.89, 1.04)	0.297			
	MR−Egger	1.06 (0.88, 1.27)	0.542	−0.009	0.008	0.238
	Weighted median	0.98 (0.88, 1.09)	0.693			
	Outlier corrected IVW	0.96 (0.89, 1.04)	0.297			
Hospitalisation for infection	IVW	1.01 (0.97, 1.06)	0.577			
	MR−Egger	0.97 (0.88, 1.07)	0.542	0.004	0.005	0.343
	Weighted median	1.00 (0.94, 1.07)	0.957			
	Outlier corrected IVW	1.01 (0.97, 1.06)	0.577			
Non−cardiovascular death	IVW	1.01 (0.95, 1.08)	0.720			
	MR−Egger	1.05 (0.91, 1.22)	0.483	−0.004	0.006	0.544
	Weighted median	1.01 (0.93, 1.10)	0.835			
	Outlier corrected IVW	1.01 (0.95, 1.08)	0.720			
End stage kidney disease (treated)	IVW	0.99 (0.74, 1.32)	0.926			
	MR−Egger	1.14 (0.58, 2.21)	0.704	−0.014	0.030	0.640
	Weighted median	1.03 (0.68, 1.53)	0.904			
	Outlier corrected IVW	0.99 (0.74, 1.32)	0.926			

FGF-23, Fibroblast growth factor 23; OR (95% CI), odds ratio (95% confidence interval); SD, standard deviation. SE, standard error; IVW=inverse variance weighted. Outlier corrected IVW used the modified Q statistic

Supplemental Figure 5: Associations between genetically-predicted FGF-23 with clinical measurements using standard methods to assess validity of instrumental variable assumptions (sensitivity analysis)

A)

Outcome	Method	Effect (95% CI) per 1−SD increase in genetically−predicted logFGF−23	p−value	Intercept	SE intercept	p−value intercept
**Carotid intimal thickness (μm)**						
Maximum	IVW	0.20 (−6.00, 6.40)	0.940			
	MR−Egger	2.70 (−11.50, 16.90)	0.708	−0.241	0.635	0.704
	Weighted median	−0.30 (−8.90, 8.30)	0.945			
	Outlier corrected IVW	0.20 (−6.00, 6.40)	0.940			
Mean	IVW	−0.60 (−5.80, 4.60)	0.810			
	MR−Egger	1.20 (−10.60, 13.10)	0.839	−0.182	0.531	0.732
	Weighted median	−1.20 (−8.60, 6.10)	0.746			
	Outlier corrected IVW	−0.60 (−5.80, 4.60)	0.810			

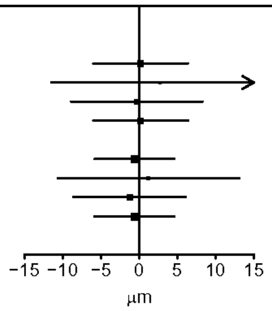

B)

Outcome	Method	Effect (95% CI) per 1−SD increase in genetically−predicted logFGF−23	p−value	Intercept	SE intercept	p−value intercept
**Left ventricular mass index (g/m^2)**						
	IVW	0.40 (0.00, 0.80)	0.068			
	MR−Egger	−0.80 (−1.70, 0.20)	0.106	0.109	0.041	0.007
	Weighted median	0.40 (−0.20, 1.00)	0.167			
	Outlier corrected IVW	0.40 (0.00, 0.80)	0.068			

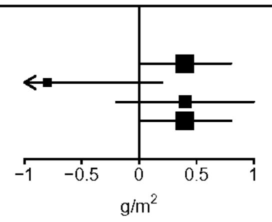

C)

Outcome	Method	Effect (95% CI) per 1−SD increase in genetically−predicted logFGF−23	p−value	Intercept	SE intercept	p−value intercept
**Bone mass (grams)**						
Android	IVW	1.30 (−0.20, 2.80)	0.091			
	MR−Egger	0.20 (−3.20, 3.70)	0.892	0.103	0.154	0.506
	Weighted median	1.20 (−0.90, 3.30)	0.264			
	Outlier corrected IVW	1.30 (−0.20, 2.80)	0.091			
Gynoid	IVW	8.60 (2.70, 14.60)	0.005			
	MR−Egger	4.70 (−9.10, 18.40)	0.505	0.384	0.611	0.530
	Weighted median	6.40 (−2.40, 15.20)	0.154			
	Outlier corrected IVW	8.60 (2.70, 14.60)	0.005			

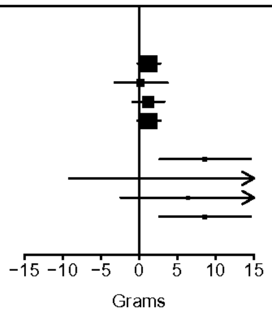

D)

Outcome	Method	Effect (95% CI) per 1−SD increase in genetically−predicted logFGF−23	p−value	Intercept	SE intercept	p−value intercept
**Bone mineral density (g/cm^3)**						
Lumbar vertebrae	IVW	0.02 (0.00, 0.05)	0.063			
	MR−Egger	0.02 (−0.04, 0.08)	0.496	0.000	0.003	0.889
	Weighted median	0.02 (−0.02, 0.05)	0.356			
	Outlier corrected IVW	0.02 (0.00, 0.05)	0.063			
Femoral neck	IVW	0.02 (0.00, 0.04)	0.067			
	MR−Egger	0.05 (0.01, 0.09)	0.028	−0.003	0.002	0.116
	Weighted median	0.01 (−0.02, 0.04)	0.545			
	Outlier corrected IVW	0.02 (0.00, 0.04)	0.067			

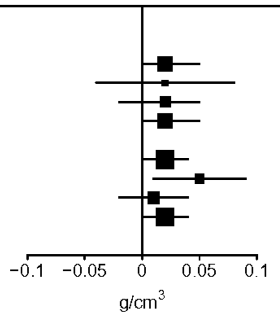

SD, standard deviation. SE, standard error; IVW=inverse variance weighted. Outlier corrected IVW used the modified Q statistic.

Supplemental Figure 6: Forest plots of effect estimates for individual SNP-FGF-23 associations and associations with key clinical outcomes

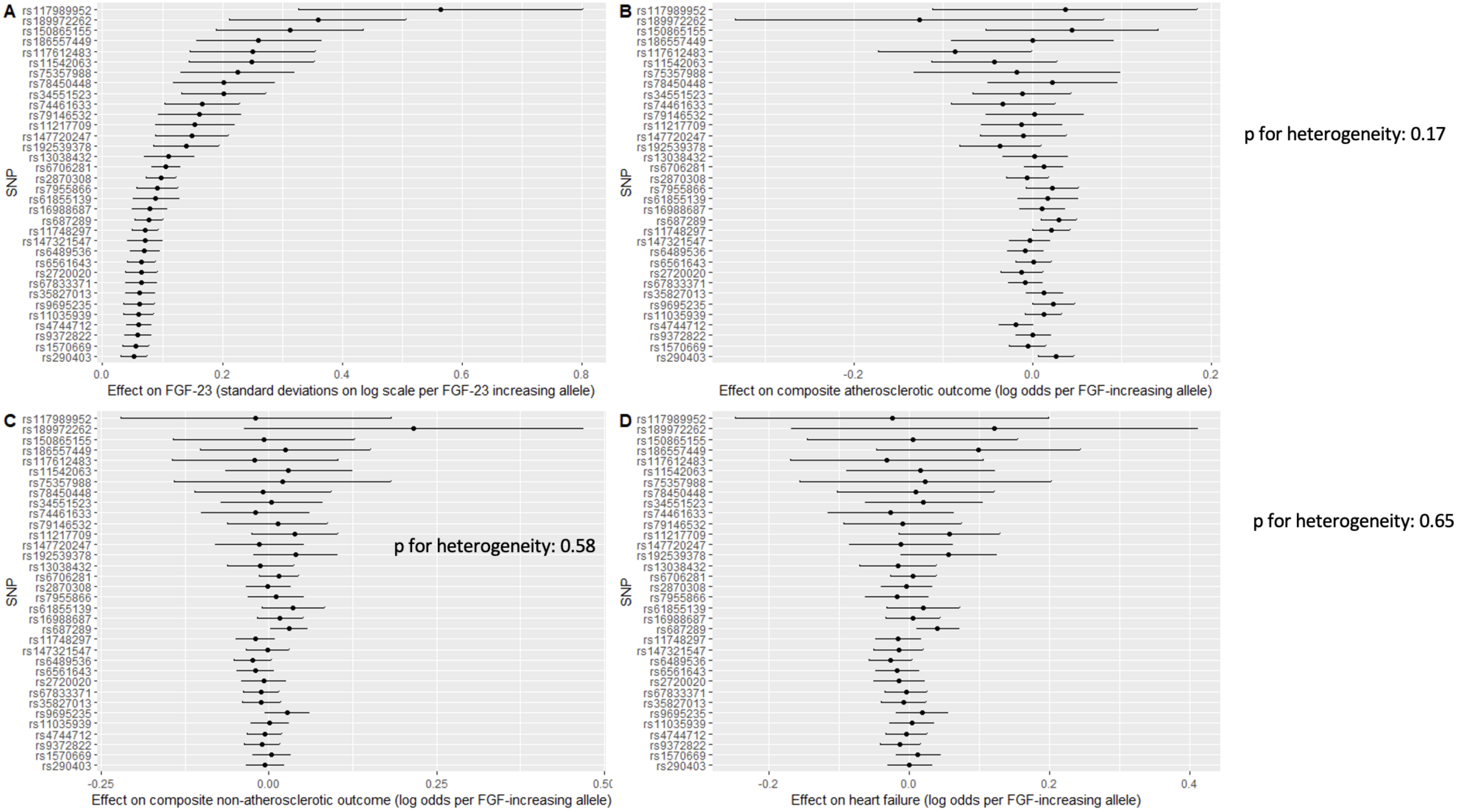

FGF-23, Fibroblast growth factor 23; Individual SNP estimates and 95% CIs for FGF-23 associations (A) and associations with the composite atherosclerotic clinical outcome (B), the composite non-atherosclerotic clinical outcome (C) and hospitalisation with heart-failure (D). Heterogeneity tests are Cochran Q tests.

Supplemental Appendix 1: OPCS-4 codes used to define the outcome "other revascularisation"

Code	Description of other revascularisation
L311	Percutaneous transluminal angioplasty of carotid artery
L313	Endovascular repair of carotid artery
L314	Percutaneous transluminal insertion of a stent into carotid artery
L318	Other specified transluminal operations on carotid artery
L319	Unspecified transluminal operations on carotid artery
L291	Replacement of carotid artery using graft
L294	Endarterectomy of carotid artery and patch repair of carotid artery
L295	Endarterectomy of carotid artery NEC
L293	Bypass to carotid artery NEC
L371	Bypass of subclavian artery NEC
L372	Endarterectomy of vertebral artery
L373	Endarterectomy of subclavian artery and patch repair of subclavian artery
L374	Endarterectomy of subclavian artery NEC
L378	Other specified of subclavian artery
L379	Unspecified reconstruction of subclavian artery
L383	Open embolectomy of subclavian artery
L391	Percutaneous transluminal angioplasty of subclavian artery
L392	Percutaneous transluminal embolectomy of subclavian artery
L395	Percutaneous transluminal insertion of stent into subclavian artery
L398	Other specified transluminal operations on subclavian artery
L399	Unspecified transluminal operations on subclavian artery
L411	Plastic repair of renal artery and end to end anastomosis of renal artery
L412	Bypass of renal artery
L413	Replantation of renal artery
L414	Endarterectomy of renal artery
L415	Translocation of branch of renal artery
L416	Patch angioplasty of renal artery
L418	Other specified reconstruction of renal artery
L419	Unspecified reconstruction of renal artery
L421	Open embolectomy of renal artery
L422	Open embolisation of renal artery
L424	Operations on aneurysm of renal artery
L428	Other specified other open operations on renal artery
L429	Unspecified other open operations on renal artery
L431	Percutaneous transluminal angioplasty of renal artery
L432	Percutaneous transluminal embolectomy of renal artery
L435	Percutaneous transluminal insertion of stent into renal artery
L438	Other specified transluminal operations on renal artery
L439	Unspecified transluminal operations on renal artery
L461	Open embolectomy of visceral branch of abdominal aorta NEC
L471	Percutaneous transluminal angioplasty of visceral branch of abdominal aorta
L474	Percutaneous transluminal insertion of stent into visceral branch of abdominal aorta NEC
L478	Other specified transluminal operations on other visceral branch of abdominal aorta
L479	Unspecified transluminal operations on other visceral branch of abdominal aorta
L481	Emergency replacement of aneurysmal common iliac artery by anastomosis of aorta to common iliac artery
L482	Emergency replacement of aneurysmal iliac artery by anastomosis of aorta to external iliac artery
L483	Emergency replacement of aneurysmal artery of leg by anastomosis of aorta to common femoral artery
L484	Emergency replacement of aneurysmal artery of leg by anastomosis of aorta to superficial femoral artery
L485	Emergency replacement of aneurysmal iliac artery by anastomosis of iliac artery to iliac artery
L486	Emergency replacement of aneurysmal artery of leg by anastomosis of iliac artery to femoral artery
L488	Other specified emergency replacement of aneurysmal iliac artery
L489	Unspecified emergency replacement of aneurysmal iliac artery
L501	Emergency bypass of common iliac artery by anastomosis of aorta to common iliac artery NEC
L502	Emergency bypass of iliac artery by anastomosis of aorta to external iliac artery NEC
L503	Emergency bypass of artery of leg by anastomosis of aorta to common femoral artery NEC
L504	Emergency bypass of artery of leg by anastomosis of aorta to deep femoral artery NEC
L505	Emergency bypass of iliac artery by anastomosis of iliac artery to iliac artery NEC
L506	Emergency bypass of artery of leg by anastomosis of iliac artery to femoral artery NEC
L508	Other specified other emergency bypass of iliac artery
L509	Unspecified other emergency bypass of iliac artery
L511	Bypass of common iliac artery by anastomosis of aorta to common iliac artery NEC
L512	Bypass of iliac artery by anastomosis of aorta to external iliac artery NEC
L513	Bypass of artery of leg by anastomosis of aorta to common femoral artery NEC
L514	Bypass of artery of leg by anastomosis of aorta to deep femoral artery NEC
L515	Bypass of iliac artery by anastomosis of iliac artery to iliac artery NEC

Code	Description of other revascularisation
L516	Bypass of artery of leg by anastomosis of iliac artery to femoral artery NEC
L518	Other specified other bypass of iliac artery
L519	Unspecified other bypass of iliac artery
L521	Endarterectomy of iliac artery and patch repair of iliac artery
L522	Endarterectomy of iliac artery NEC
L528	Other specified reconstruction of iliac artery
L529	Unspecified reconstruction of iliac artery
L531	Repair of iliac artery NEC
L532	Open embolectomy of iliac artery
L533	Operations on aneurysm of iliac artery NEC
L538	Other specified open operations on iliac artery
L539	Unspecified other open operations on iliac artery
L541	Percutaneous transluminal angioplasty of iliac artery
L542	Percutaneous transluminal embolectomy of iliac artery
L544	Percutaneous transluminal insertion of stent into iliac artery
L548	Other specified transluminal operations on iliac artery
L549	Unspecified transluminal operations on iliac artery
L561	Emergency replacement of aneurysmal femoral artery by anastomosis of femoral artery to femoral artery
L562	Emergency replacement of aneurysmal femoral artery by anastomosis of femoral artery to popliteal artery using prosthesis
L563	Emergency replacement of aneurysmal femoral artery by anastomosis of femoral artery to popliteal artery using vein graft
L564	Emergency replacement of aneurysmal femoral artery by anastomosis of femoral artery to tibial artery using prosthesis
L565	Emergency replacement of aneurysmal femoral artery by anastomosis of femoral artery to tibial artery using vein graft
L566	Emergency replacement of aneurysmal femoral artery by anastomosis of femoral artery to peroneal artery using prosthesis
L567	Emergency replacement of aneurysmal femoral artery by anastomosis of femoral artery to peroneal artery using vein graft
L568	Other specified emergency replacement of aneurysmal femoral artery
L569	Unspecified emergency replacement of aneurysmal femoral artery
L571	Replacement of aneurysmal femoral artery by anastomosis of femoral artery to femoral artery NEC
L572	Replacement of aneurysmal femoral artery by anastomosis of femoral artery to popliteal artery using prosthesis NEC
L573	Replacement of aneurysmal femoral artery by anastomosis of femoral artery to popliteal artery using vein graft NEC
L574	Replacement of aneurysmal femoral artery by anastomosis of femoral artery to tibial artery using prosthesis NEC
L575	Replacement of aneurysmal femoral artery by anastomosis of femoral artery to tibial artery using vein graft NEC
L576	Replacement of aneurysmal femoral artery by anastomosis of femoral artery to peroneal artery using prosthesis NEC
L577	Replacement of aneurysmal femoral artery by anastomosis of femoral artery to peroneal artery using vein graft NEC
L578	Other specified other replacement of aneurysmal femoral artery
L579	Unspecified other replacement of aneurysmal femoral artery
L581	Emergency bypass of femoral artery by anastomosis of femoral artery to femoral artery NEC
L582	Emergency bypass of femoral artery by anastomosis of femoral artery to popliteal artery using prosthesis NEC
L583	Emergency bypass of femoral artery by anastomosis of femoral artery to popliteal artery using vein graft NEC
L584	Emergency bypass of femoral artery by anastomosis of femoral artery to tibial artery using prosthesis NEC
L585	Emergency bypass of femoral artery by anastomosis of femoral artery to tibial artery using vein graft NEC
L586	Emergency bypass of femoral artery by anastomosis of femoral artery to peroneal artery using prosthesis NEC
L587	Emergency bypass of femoral artery by anastomosis of femoral artery to peroneal artery using vein graft NEC
L588	Other specified other emergency bypass of femoral artery
L589	Unspecified other emergency bypass of femoral artery
L591	Bypass of femoral artery by anastomosis of femoral artery to femoral artery NEC
L592	Bypass of femoral artery by anastomosis of femoral artery to popliteal artery using prosthesis NEC
L593	Bypass of femoral artery by anastomosis of femoral artery to popliteal artery using vein graft NEC
L594	Bypass of femoral artery by anastomosis of femoral artery to tibial artery using prosthesis NEC
L595	Bypass of femoral artery by anastomosis of femoral artery to tibial artery using vein graft NEC
L596	Bypass of femoral artery by anastomosis of femoral artery to peroneal artery using prosthesis NEC
L597	Bypass of femoral artery by anastomosis of femoral artery to peroneal artery using vein graft NEC
L598	Other specified other bypass of femoral artery
L599	Unspecified other bypass of femoral artery
L601	Endarterectomy of femoral artery and patch repair of femoral artery
L602	Endarterectomy of femoral artery NEC
L603	Profundoplasty of femoral artery and patch repair of deep femoral artery
L604	Profundoplasty of femoral artery NEC
L608	Other specified reconstruction of femoral artery
L609	Unspecified reconstruction of femoral artery
L621	Repair of femoral artery NEC
L622	Repair of femoral artery NEC
L624	Operations on aneurysm of femoral artery NEC
L628	Other specified other open operations on femoral artery
L629	Unspecified other open operations on femoral artery
L631	Percutaneous transluminal angioplasty of femoral artery

Code	Description of other revascularisation
L632	Percutaneous transluminal embolectomy of femoral artery
L633	Percutaneous transluminal embolisation of femoral artery
L635	Percutaneous transluminal insertion of stent into femoral artery
L638	Other specified transluminal operations on femoral artery
L639	Unspecified transluminal operations on femoral artery
L661	Percutaneous transluminal arterial thrombolysis and reconstruction
L662	Percutaneous transluminal stent reconstruction of artery
L665	Percutaneous transluminal balloon angioplasty of artery
L667	Percutaneous transluminal placement of peripheral stent in artery
L668	Other specified other therapeutic transluminal operations on artery
L669	Unspecified other therapeutic transluminal operations on artery
L718	Other specified therapeutic transluminal operations on other artery
L719	Unspecified therapeutic transluminal operations on other artery
L681	Endarterectomy and patch repair of artery NEC
L682	Endarterectomy NEC

Supplemental Appendix 2: ICD-10 diagnostic codes used to define hospitalisation for infection

Code	Description of infection
A01	Typhoid and paratyphoid fevers
A010	Typhoid fever
A011	Paratyphoid fever A
A012	Paratyphoid fever B
A013	Paratyphoid fever C
A014	Paratyphoid fever, unspecified
A021	Salmonella sepsis
A047	Enterocolitis due to Clostridium difficile
A06	Amebiasis
A060	Acute amebic dysentery
A061	Chronic intestinal amebiasis
A062	Amebic nondysenteric colitis
A063	Ameboma of intestine
A064	Amebic liver abscess
A065	Amebic lung abscess
A066	Amebic brain abscess
A067	Cutaneous amebiasis
A068	Amebic infection of other sites
A069	Amebiasis, unspecified
A15	Respiratory tuberculosis
A150	Tuberculosis of lung
A154	Tuberculosis of intrathoracic lymph nodes
A155	Tuberculosis of larynx, trachea and bronchus
A156	Tuberculous pleurisy
A157	Primary respiratory tuberculosis
A158	Other respiratory tuberculosis
A159	Respiratory tuberculosis unspecified
A17	Tuberculosis of nervous system
A170	Tuberculous meningitis
A171	Meningeal tuberculoma
A178	Other tuberculosis of nervous system
A179	Tuberculosis of nervous system, unspecified
A18	Tuberculosis of other organs
A180	Tuberculosis of bones and joints
A181	Tuberculosis of genitourinary system
A182	Tuberculous peripheral lymphadenopathy
A183	Tuberculosis of intestines, peritoneum and mesenteric glands
A184	Tuberculosis of skin and subcutaneous tissue
A185	Tuberculosis of eye
A186	Tuberculosis of (inner) (middle) ear
A187	Tuberculosis of adrenal glands
A188	Tuberculosis of other specified organs
A19	Miliary tuberculosis
A190	Acute miliary tuberculosis of a single specified site
A191	Acute miliary tuberculosis of multiple sites
A192	Acute miliary tuberculosis, unspecified
A198	Other miliary tuberculosis
A199	Miliary tuberculosis, unspecified
A20	Plague
A200	Bubonic plague
A201	Cellulocutaneous plague
A202	Pneumonic plague
A203	Plague meningitis
A207	Septicemic plague
A208	Other forms of plague
A209	Plague, unspecified
A21	Tularemia
A210	Ulceroglandular tularemia
A211	Oculoglandular tularemia
A212	Pulmonary tularemia
A213	Gastrointestinal tularemia
A217	Generalized tularemia
A218	Other forms of tularemia
A219	Tularemia, unspecified
A22	Anthrax
A220	Cutaneous anthrax
A221	Pulmonary anthrax

Code	Description of infection
A222	Gastrointestinal anthrax
A227	Anthrax sepsis
A228	Other forms of anthrax
A229	Anthrax, unspecified
A23	Brucellosis
A230	Brucellosis due to Brucella melitensis
A231	Brucellosis due to Brucella abortus
A232	Brucellosis due to Brucella suis
A233	Brucellosis due to Brucella canis
A238	Other brucellosis
A239	Brucellosis, unspecified
A24	Glanders and melioidosis
A240	Glanders
A241	Acute and fulminating melioidosis
A242	Subacute and chronic melioidosis
A243	Other melioidosis
A249	Melioidosis, unspecified
A25	Rat-bite fevers
A250	Spirillosis
A251	Streptobacillosis
A259	Rat-bite fever, unspecified
A26	Erysipeloid
A260	Cutaneous erysipeloid
A267	Erysipelothrix sepsis
A268	Other forms of erysipeloid
A269	Erysipeloid, unspecified
A27	Leptospirosis
A270	Leptospirosis icterohemorrhagica
A278	Other forms of leptospirosis
A279	Leptospirosis, unspecified
A28	Other zoonotic bacterial diseases, not elsewhere classified
A280	Pasteurellosis
A281	Cat-scratch disease
A282	Extraintestinal yersiniosis
A288	Other specified zoonotic bacterial diseases, not elsewhere classified
A289	Zoonotic bacterial disease, unspecified
A30	Leprosy [Hansen's disease]
A300	Indeterminate leprosy
A301	Tuberculoid leprosy
A302	Borderline tuberculoid leprosy
A303	Borderline leprosy
A304	Borderline lepromatous leprosy
A305	Lepromatous leprosy
A308	Other forms of leprosy
A309	Leprosy, unspecified
A31	Infection due to other mycobacteria
A310	Pulmonary mycobacterial infection
A311	Cutaneous mycobacterial infection
A312	Disseminated mycobacterium avium-intracellulare complex (DMAC)
A318	Other mycobacterial infections
A319	Mycobacterial infection, unspecified
A32	Listeriosis
A320	Cutaneous listeriosis
A321	Listerial meningitis and meningoencephalitis
A327	Listerial sepsis
A328	Other forms of listeriosis
A329	Listeriosis, unspecified
A33	Tetanus neonatorum
A34	Obstetrical tetanus
A35	Other tetanus
A36	Diphtheria
A360	Pharyngeal diphtheria
A361	Nasopharyngeal diphtheria
A362	Laryngeal diphtheria
A363	Cutaneous diphtheria
A368	Other diphtheria
A369	Diphtheria, unspecified
A37	Whooping cough

Code	Description of infection
A370	Whooping cough due to Bordetella pertussis
A371	Whooping cough due to Bordetella parapertussis
A378	Whooping cough due to other Bordetella species
A379	Whooping cough, unspecified species
A38	Scarlet fever
A380	Scarlet fever with otitis media
A381	Scarlet fever with myocarditis
A388	Scarlet fever with other complications
A389	Scarlet fever, uncomplicated
A39	Meningococcal infection
A390	Meningococcal meningitis
A391	Waterhouse-Friderichsen syndrome
A392	Acute meningococcemia
A393	Chronic meningococcemia
A394	Meningococcemia, unspecified
A395	Meningococcal heart disease
A398	Other meningococcal infections
A399	Meningococcal infection, unspecified
A40	Streptococcal sepsis
A400	Sepsis due to streptococcus, group A
A401	Sepsis due to streptococcus, group B
A403	Sepsis due to Streptococcus pneumoniae
A408	Other streptococcal sepsis
A409	Streptococcal sepsis, unspecified
A41	Other sepsis
A410	Sepsis due to Staphylococcus aureus
A411	Sepsis due to other specified staphylococcus
A412	Sepsis due to unspecified staphylococcus
A413	Sepsis due to Hemophilus influenzae
A414	Sepsis due to anaerobes
A415	Sepsis due to other Gram-negative organisms
A418	Other specified sepsis
A419	Sepsis, unspecified organism
A42	Actinomycosis
A420	Pulmonary actinomycosis
A421	Abdominal actinomycosis
A422	Cervicofacial actinomycosis
A427	Actinomycotic sepsis
A428	Other forms of actinomycosis
A429	Actinomycosis, unspecified
A43	Nocardiosis
A430	Pulmonary nocardiosis
A431	Cutaneous nocardiosis
A438	Other forms of nocardiosis
A439	Nocardiosis, unspecified
A44	Bartonellosis
A440	Systemic bartonellosis
A441	Cutaneous and mucocutaneous bartonellosis
A448	Other forms of bartonellosis
A449	Bartonellosis, unspecified
A46	Erysipelas
A480	Gas gangrene
A481	Legionnaires' disease
A482	Nonpneumonic Legionnaires' disease [Pontiac fever]
A483	Toxic shock syndrome
A484	Brazilian purpuric fever
A490	Staphylococcal infection, unspecified site
A491	Streptococcal infection, unspecified site
A492	Hemophilus influenzae infection, unspecified site
A493	Mycoplasma infection, unspecified site
A50	Congenital syphilis
A500	Early congenital syphilis, symptomatic
A501	Early congenital syphilis, latent
A502	Early congenital syphilis, unspecified
A503	Late congenital syphilitic oculopathy
A504	Late congenital neurosyphilis [juvenile neurosyphilis]
A505	Other late congenital syphilis, symptomatic
A506	Late congenital syphilis, latent

Code	Description of infection
A507	Late congenital syphilis, unspecified
A509	Congenital syphilis, unspecified
A51	Early syphilis
A510	Primary genital syphilis
A511	Primary anal syphilis
A512	Primary syphilis of other sites
A513	Secondary syphilis of skin and mucous membranes
A514	Other secondary syphilis
A515	Early syphilis, latent
A519	Early syphilis, unspecified
A52	Late syphilis
A520	Cardiovascular and cerebrovascular syphilis
A521	Symptomatic neurosyphilis
A522	Asymptomatic neurosyphilis
A523	Neurosyphilis, unspecified
A527	Other symptomatic late syphilis
A528	Late syphilis, latent
A529	Late syphilis, unspecified
A53	Other and unspecified syphilis
A530	Latent syphilis, unspecified as early or late
A539	Syphilis, unspecified
A54	Gonococcal infection
A540	Gonococcal infection of lower genitourinary tract without periurethral or accessory gland abscess
A541	Gonococcal infection of lower genitourinary tract with periurethral and accessory gland abscess
A542	Gonococcal pelviperitonitis and other gonococcal genitourinary infection
A543	Gonococcal infection of eye
A544	Gonococcal infection of musculoskeletal system
A545	Gonococcal pharyngitis
A546	Gonococcal infection of anus and rectum
A548	Other gonococcal infections
A549	Gonococcal infection, unspecified
A55	Chlamydial lymphogranuloma (venereum)
A56	Other sexually transmitted chlamydial diseases
A560	Chlamydial infection of lower genitourinary tract
A561	Chlamydial infection of pelviperitoneum and other genitourinary organs
A562	Chlamydial infection of genitourinary tract, unspecified
A563	Chlamydial infection of anus and rectum
A564	Chlamydial infection of pharynx
A568	Sexually transmitted chlamydial infection of other sites
A57	Chancroid
A58	Granuloma inguinale
A59	Trichomoniasis
A590	Urogenital trichomoniasis
A598	Trichomoniasis of other sites
A599	Trichomoniasis, unspecified
A65	Nonvenereal syphilis
A66	Yaws
A660	Initial lesions of yaws
A661	Multiple papillomata and wet crab yaws
A662	Other early skin lesions of yaws
A663	Hyperkeratosis of yaws
A664	Gummata and ulcers of yaws
A665	Gangosa
A666	Bone and joint lesions of yaws
A667	Other manifestations of yaws
A668	Latent yaws
A669	Yaws, unspecified
A67	Pinta [carate]
A670	Primary lesions of pinta
A671	Intermediate lesions of pinta
A672	Late lesions of pinta
A673	Mixed lesions of pinta
A679	Pinta, unspecified
A68	Relapsing fevers
A680	Louse-borne relapsing fever
A681	Tick-borne relapsing fever
A689	Relapsing fever, unspecified
A69	Other spirochetal infections

Code	Description of infection
A690	Necrotizing ulcerative stomatitis
A691	Other Vincent's infections
A692	Lyme disease
A698	Other specified spirochetal infections
A699	Spirochetal infection, unspecified
A70	Chlamydia psittaci infections
A71	Trachoma
A710	Initial stage of trachoma
A711	Active stage of trachoma
A719	Trachoma, unspecified
A74	Other diseases caused by chlamydiae
A740	Chlamydial conjunctivitis
A748	Other chlamydial diseases
A749	Chlamydial infection, unspecified
A75	Typhus fever
A750	Epidemic louse-borne typhus fever due to Rickettsia prowazekii
A751	Recrudescent typhus [Brill's disease]
A752	Typhus fever due to Rickettsia typhi
A753	Typhus fever due to Rickettsia tsutsugamushi
A759	Typhus fever, unspecified
A77	Spotted fever [tick-borne rickettsioses]
A770	Spotted fever due to Rickettsia rickettsii
A771	Spotted fever due to Rickettsia conorii
A772	Spotted fever due to Rickettsia siberica
A773	Spotted fever due to Rickettsia australis
A774	Ehrlichiosis
A778	Other spotted fevers
A779	Spotted fever, unspecified
A78	Q fever
A79	Other rickettsioses
A790	Trench fever
A791	Rickettsialpox due to Rickettsia akari
A798	Other specified rickettsioses
A799	Rickettsiosis, unspecified
B58	Toxoplasmosis
B580	Toxoplasma oculopathy
B581	Toxoplasma hepatitis
B582	Toxoplasma meningoencephalitis
B583	Pulmonary toxoplasmosis
B588	Toxoplasmosis with other organ involvement
B589	Toxoplasmosis, unspecified
B59	Pneumocystosis
B600	Babesiosis
B601	Acanthamebiasis
B602	Naegleriasis
B90	Sequelae of tuberculosis
B900	Sequelae of central nervous system tuberculosis
B901	Sequelae of genitourinary tuberculosis
B902	Sequelae of tuberculosis of bones and joints
B908	Sequelae of tuberculosis of other organs
B909	Sequelae of respiratory and unspecified tuberculosis
B92	Sequelae of leprosy
B940	Sequelae of trachoma
B95	Streptococcus, Staphylococcus, and Enterococcus as the cause of diseases classified elsewhere
B950	Streptococcus, group A, as the cause of diseases classified elsewhere
B951	Streptococcus, group B, as the cause of diseases classified elsewhere
B952	Enterococcus as the cause of diseases classified elsewhere
B953	Streptococcus pneumoniae as the cause of diseases classified elsewhere
B954	Other streptococcus as the cause of diseases classified elsewhere
B955	Unspecified streptococcus as the cause of diseases classified elsewhere
B956	Staphylococcus aureus as the cause of diseases classified elsewhere
B957	Other staphylococcus as the cause of diseases classified elsewhere
B958	Unspecified staphylococcus as the cause of diseases classified elsewhere
B960	Mycoplasma pneumoniae [M. pneumoniae] as the cause of diseases classified elsewhere
B961	Klebsiella pneumoniae [K. pneumoniae] as the cause of diseases classified elsewhere
B963	Hemophilus influenzae [H. influenzae] as the cause of diseases classified elsewhere
B964	Proteus (mirabilis) (morganii) as the cause of diseases classified elsewhere
B965	Pseudomonas (aeruginosa) (mallei) (pseudomallei) as the cause of diseases classified elsewhere

Code	Description of infection
B966	Bacteroides fragilis [B. fragilis] as the cause of diseases classified elsewhere
B967	Clostridium perfringens [C. perfringens] as the cause of diseases classified elsewhere
B968	Other specified bacterial agents as the cause of diseases classified elsewhere
D733	Abscess of spleen
E321	Abscess of thymus
G00	Bacterial meningitis, not elsewhere classified
G000	Hemophilus meningitis
G001	Pneumococcal meningitis
G002	Streptococcal meningitis
G003	Staphylococcal meningitis
G008	Other bacterial meningitis
G009	Bacterial meningitis, unspecified
G01	Meningitis in bacterial diseases classified elsewhere
G042	Bacterial meningoencephalitis and meningomyelitis, not elsewhere classified
G06	Intracranial and intraspinal abscess and granuloma
G060	Intracranial abscess and granuloma
G061	Intraspinal abscess and granuloma
G062	Extradural and subdural abscess, unspecified
G07	Intracranial and intraspinal abscess and granuloma in diseases classified elsewhere
H050	Acute inflammation of orbit
H602	Malignant otitis externa
H700	Acute mastoiditis
H701	Chronic mastoiditis
H702	Petrositis
H708	Other mastoiditis and related conditions
H709	Unspecified mastoiditis
H750	Mastoiditis in infectious and parasitic diseases classified elsewhere
I00	Rheumatic fever without heart involvement
I01	Rheumatic fever with heart involvement
I010	Acute rheumatic pericarditis
I011	Acute rheumatic endocarditis
I012	Acute rheumatic myocarditis
I018	Other acute rheumatic heart disease
I019	Acute rheumatic heart disease, unspecified
I02	Rheumatic chorea
I020	Rheumatic chorea with heart involvement
I029	Rheumatic chorea without heart involvement
I33	Acute and subacute endocarditis
I330	Acute and subacute infective endocarditis
I339	Acute and subacute endocarditis, unspecified
I38	Endocarditis, valve unspecified
I39	Endocarditis and heart valve disorders in diseases classified elsewhere
I76	Septic arterial embolism
I96	Gangrene, not elsewhere classified
J020	Streptococcal pharyngitis
J030	Streptococcal tonsillitis
J13	Pneumonia due to Streptococcus pneumoniae
J14	Pneumonia due to Hemophilus influenzae
J15	Bacterial pneumonia, not elsewhere classified
J150	Pneumonia due to Klebsiella pneumoniae
J151	Pneumonia due to Pseudomonas
J152	Pneumonia due to staphylococcus
J153	Pneumonia due to streptococcus, group B
J154	Pneumonia due to other streptococci
J155	Pneumonia due to Escherichia coli
J156	Pneumonia due to other Gram-negative bacteria
J157	Pneumonia due to Mycoplasma pneumoniae
J158	Pneumonia due to other specified bacteria
J159	Unspecified bacterial pneumonia
J16	Pneumonia due to other infectious organisms, not elsewhere classified
J160	Chlamydial pneumonia
J168	Pneumonia due to other specified infectious organisms
J17	Pneumonia in diseases classified elsewhere
J18	Pneumonia, unspecified organism
J180	Bronchopneumonia, unspecified organism
J181	Lobar pneumonia, unspecified organism
J182	Hypostatic pneumonia, unspecified organism
J188	Other pneumonia, unspecified organism

Code	Description of infection
J189	Pneumonia, unspecified organism
J36	Peritonsillar abscess
J390	Retropharyngeal and parapharyngeal abscess
J391	Other abscess of pharynx
J85	Abscess of lung and mediastinum
J850	Gangrene and necrosis of lung
J851	Abscess of lung with pneumonia
J852	Abscess of lung without pneumonia
J853	Abscess of mediastinum
J86	Pyothorax
J860	Pyothorax with fistula
J869	Pyothorax without fistula
K046	Periapical abscess with sinus
K047	Periapical abscess without sinus
K113	Abscess of salivary gland
K122	Cellulitis and abscess of mouth
K35	Acute appendicitis
K352	Acute appendicitis with generalized peritonitis
K353	Acute appendicitis with localized peritonitis
K358	Other and unspecified acute appendicitis
K36	Other appendicitis
K37	Unspecified appendicitis
K401	Bilateral inguinal hernia, with gangrene
K404	Unilateral inguinal hernia, with gangrene
K411	Bilateral femoral hernia, with gangrene
K414	Unilateral femoral hernia, with gangrene
K421	Umbilical hernia with gangrene
K431	Incisional hernia with gangrene
K434	Parastomal hernia with gangrene
K437	Other and unspecified ventral hernia with gangrene
K441	Diaphragmatic hernia with gangrene
K451	Other specified abdominal hernia with gangrene
K461	Unspecified abdominal hernia with gangrene
K570	Diverticulitis of small intestine with perforation and abscess
K572	Diverticulitis of large intestine with perforation and abscess
K574	Diverticulitis of both small and large intestine with perforation and abscess
K578	Diverticulitis of intestine, part unspecified, with perforation and abscess
K630	Abscess of intestine
K650	Generalized (acute) peritonitis
K651	Peritoneal abscess
K652	Spontaneous bacterial peritonitis
K653	Choleperitonitis
K658	Other peritonitis
K659	Peritonitis, unspecified
K681	Retroperitoneal abscess
K750	Abscess of liver
K800	Calculus of gallbladder with acute cholecystitis
K801	Calculus of gallbladder with other cholecystitis
K803	Calculus of bile duct with cholangitis
K804	Calculus of bile duct with cholecystitis
K806	Calculus of gallbladder and bile duct with cholecystitis
K81	Cholecystitis
K810	Acute cholecystitis
K811	Chronic cholecystitis
K812	Acute cholecystitis with chronic cholecystitis
K819	Cholecystitis, unspecified
K830	Cholangitis
K901	Tropical sprue
L00	Staphylococcal scalded skin syndrome
M00	Pyogenic arthritis
M000	Staphylococcal arthritis and polyarthritis
M001	Pneumococcal arthritis and polyarthritis
M002	Other streptococcal arthritis and polyarthritis
M008	Arthritis and polyarthritis due to other bacteria
M009	Pyogenic arthritis, unspecified
M01	Direct infections of joint in infectious and parasitic diseases classified elsewhere
M01X	Direct infection of joint in infectious and parasitic diseases classified elsewhere
M462	Osteomyelitis of vertebra

Code	Description of infection
M463	Infection of intervertebral disc (pyogenic)
M650	Abscess of tendon sheath
M651	Other infective (teno)synovitis
M726	Necrotizing fasciitis
M86	Osteomyelitis
M860	Acute hematogenous osteomyelitis
M861	Other acute osteomyelitis
M862	Subacute osteomyelitis
M863	Chronic multifocal osteomyelitis
M864	Chronic osteomyelitis with draining sinus
M865	Other chronic hematogenous osteomyelitis
M866	Other chronic osteomyelitis
M868	Other osteomyelitis
M869	Osteomyelitis, unspecified
N10	Acute pyelonephritis
N151	Renal and perinephric abscess
N300	Acute cystitis
N303	Trigonitis
N340	Urethral abscess
N390	Urinary tract infection, site not specified
N410	Acute prostatitis
N412	Abscess of prostate
N431	Infected hydrocele
N454	Abscess of epididymis or testis
N493	Fournier gangrene
N70	Salpingitis and oophoritis
N700	Acute salpingitis and oophoritis
N701	Chronic salpingitis and oophoritis
N709	Salpingitis and oophoritis, unspecified
N71	Inflammatory disease of uterus, except cervix
N710	Acute inflammatory disease of uterus
N711	Chronic inflammatory disease of uterus
N719	Inflammatory disease of uterus, unspecified
N72	Inflammatory disease of cervix uteri
N730	Acute parametritis and pelvic cellulitis
N731	Chronic parametritis and pelvic cellulitis
N732	Unspecified parametritis and pelvic cellulitis
N733	Female acute pelvic peritonitis
N734	Female chronic pelvic peritonitis
N735	Female pelvic peritonitis, unspecified
N738	Other specified female pelvic inflammatory diseases
N739	Female pelvic inflammatory disease, unspecified
N74	Female pelvic inflammatory disorders in diseases classified elsewhere
N980	Infection associated with artificial insemination
T802	Infections following infusion, transfusion and therapeutic injection
T826	Infection and inflammatory reaction due to cardiac valve prosthesis
T827	Infection and inflammatory reaction due to other cardiac and vascular devices, implants and grafts
T835	Infection and inflammatory reaction due to prosthetic device, implant and graft in urinary system
T836	Infection and inflammatory reaction due to prosthetic device, implant and graft in genital tract
T845	Infection and inflammatory reaction due to internal joint prosthesis
T846	Infection and inflammatory reaction due to internal fixation device
T847	Infection and inflammatory reaction due to other internal orthopedic prosthetic devices, implants and grafts
T857	Infection and inflammatory reaction due to other internal prosthetic devices, implants and grafts
T874	Infection of amputation stump
T875	Necrosis of amputation stump
